# A socio-technical framework for cyber-resilience in hybrid oil-renewable energy grids

**DOI:** 10.1186/s42400-026-00619-x

**Published:** 2026-07-01

**Authors:** Bryan Anderson, Gahangir Hossain

**Affiliations:** 1 Department of Information Science, University of North Texas, 1155 Union Circle, Denton, TX 76203 USA; 2 Department of Data Science, University of North Texas, Denton, TX USA

## Abstract

Combining traditional oil infrastructure with distributed renewable energy resources (DERs), including critical grid-edge assets like electric vehicle (EV) charging infrastructure that link transportation and power systems, has created hybrid energy systems that are more connected and digitally complex. These systems integrate legacy operational technologies (OT), such as SCADA, with decentralized, IoT-enabled assets that often rely on cloud-based analytics and remote connectivity. This conjunction introduces cybersecurity risks by linking historically isolated OT environments to modern, internet-exposed components, creating more entry points, inconsistent security baselines, and new attack surfaces. The resulting vulnerabilities extend beyond traditional IT-centric threat models. Conventional cybersecurity strategies, which prioritize technical controls in isolation, often fail to address systemic risks stemming from institutional fragmentation, regulatory gaps, and third-party dependencies. This study introduces a Socio-Technical Resilience Framework grounded in the Systems-Theoretic Accident Model and Processes (STAMP) and Socio-Technical Systems (STS) theory to access and mitigate cyber risks in hybrid grids. Through comparative case studies of the Colonial Pipeline ransomware event and cyber disruptions in European DER infrastructure, the paper finds that fragmented coordination, isolated threat intelligence, and weak human-system integration significantly amplify cyber impacts that undermine static, perimeter-based defense models. The study contributes a layered framework for cyber resilience that operates across three domains: (1) technical (via zero-trust architecture and distributed anomaly detection), (2) organizational (through shared situational awareness and cognitive decision-support tools), and (3) governance (by leveraging federated threat intelligence and regulatory harmonization). This multi-domain approach enhances both operational flexibility and long-term sustainability. Ultimately, the paper demonstrates that enduring cyber resilience in hybrid energy systems requires more than patching vulnerabilities. It demands a systemic rethinking of control, coordination, and design. The framework and findings offer a forward-looking roadmap for securing the energy sector against evolving threats in an era of distributed complexity.

## Introduction

Over the past couple of decades, global energy systems have attempted to undergo a rapid transition toward hybrid models that combine traditional oil-based infrastructure with distributed renewable energy sources such as solar photovoltaics, wind turbines, battery storage, and smart meters. This is largely due to the urgent need to reduce carbon emissions, enhance energy resilience, and capitalize on the declining cost of renewable technologies and digital infrastructure. These systems are increasingly digitized, integrating legacy control platforms such as Supervisory Control and Data Acquisition (SCADA) systems, with newer technologies like edge computing, cloud-based analytics, and IoT-enabled grid devices. The result highlights a cyber-physical ecosystem that promises greater efficiency, lower emissions, and broader energy accessibility. According to recent studies on hybrid grid development, these systems now account for a significant share of new grid deployments, especially in industrialized economies (Khan et al. 2025).

In parallel, the merging of operational technology (OT) and information technology (IT) has significantly expanded the cyber-attack surface for critical energy infrastructure by exposing traditionally isolated industrial control systems that were once protected through physical separation, to external networks, cloud platforms, and third-party interfaces. While legacy systems operated in physically isolated or manually controlled environments, today’s hybrid grids rely on remote connectivity, real-time automation, and third-party digital services to coordinate energy distribution and demand forecasting. As the complexity and interdependence of these systems grow, so do the opportunities for cyber attackers to exploit vulnerabilities. Incidents such as the Colonial Pipeline ransomware attack (2021) and DER-targeted disruptions across European smart grids (contextualized by U.S. DER vulnerability analysis) underscore the growing risks (Easterly and Fanning 2023; Gorman et al. 2024; U.S. Department of Energy, n.d.). These events did not merely exploit software vulnerabilities; they revealed weaknesses in cross-organizational coordination, slow incident response, and fragmented regulatory authority (Lubin 2023). These vulnerabilities extend to EV charging networks, a critical example of grid-edge assets where disruptions can affect both transportation and energy stability.

This paper argues that cybersecurity risk in hybrid energy systems is no longer just a technical challenge but more of a socio-technical problem that demands an integrated, multi-layered approach. Conventional strategies like patching, segmentation, or perimeter defense remain essential but insufficient. Recent findings from the U.S. Department of Energy and the Cybersecurity and Infrastructure Security Agency confirm that a substantial portion of energy-sector cyber incidents stem from misaligned workflows, poor interagency communication, and insecure vendor devices, factors that are often overlooked by purely technical frameworks (U.S. Department of Energy [DOE] 2022; Cybersecurity and Infrastructure Security Agency [CISA] 2023).

Despite the increasing urgency of this issue, a critical gap persists in current academic and policy literature: there is no widely adopted framework that explicitly links technical infrastructure, human-centered operations, and governance-level coordination for securing hybrid oil-renewable energy grids. Although some resilience models emphasize “resilience-by-design” or anomaly detection, others explore human factors or compliance protocols although these efforts remain largely independent. Furthermore, many existing tools such as STAMP, Bow-Tie risk models, NERC CIP, or the NIST SP 800-82r3 offer limited guidance for dynamic, distributed, and multi-stakeholder environments like those found in DER-integrated grids (Stouffer et al. 2023).

This gap leads to a central research question: How can socio-technical resilience be systematically designed to protect hybrid oil-renewable energy grids from cyber threats?

To answer this, the paper introduces a multi-layer Socio-Technical Resilience Framework that integrates technical systems, organizational readiness, and institutional governance using concepts from STS theory and the STAMP model.

## Literature review

### Cyber threats in oil and industrial control systems (ICS)

Early SCADA surveys identified persistent weaknesses in legacy pipeline networks, such as unencrypted serial links, default credentials, and weak role-based access (Leveson 2003). NIST SP 800–82 Rev. 3 formalized these issues, noting insecure remote-access gateways and authentication gaps in refineries and offshore rigs (Igure et al. 2006). Mohammed et al. (2022) quantified the risk on 14 offshore platforms, reporting a mean time-to-compromise of less than 15 min once an attacker reached the process network. Post-mortem analyses of the 2021 Colonial Pipeline ransomware event show that a purely IT-side intrusion triggered an OT shutdown because the company lacked an approved contingency bridge for billing data (U.S. Department of Energy [DOE], n.d.). A two-year follow-up by CISA details how joint pipeline–operator drills have only recently addressed that gap (Easterly and Fanning 2023). Research on emerging threats such as the Iran-linked “CyberAv3ngers,” which breached U.S. water and gas ICS in late 2024, confirms that state actors are increasingly weaponizing custom OT malware to exploit the same control-layer flaws (Greenberg 2025).

Synthesis 1: The oil–ICS literature converges on a handful of persistent control-layer flaws. Recent work emphasizes that even IT-side ransomware can force an OT outage when governance and fallback procedures are weak.

### Cybersecurity challenges in renewable & distributed energy resources (DERs)

The DOE’s 2022 sector report catalogued 37 inverter and smart meter CVEs, linking 61% of them to unsigned firmware or unauthenticated update channels (U.S. Department of Energy [DOE] 2022). Recent national guidance, such as the i2X Transmission Interconnection Roadmap, underscores the growing urgency of secure, scalable coordination between DER assets and transmission operators (Gorman et al. 2024). Chen et al. proposed a five-layer attack surface taxonomy (devices, fieldbus, aggregation, cloud, and market), showing that vendor patch latency (< 30 days) is the dominant residual risk for high-penetration solar (Davis 2021).

Applied Energy experiments used an autoencoder to detect micro-inverter anomalies with a 97% F1-score, though performance declined when models were not retrained for brand-specific signatures (Zideh et al. 2024). A 2024 field deployment study by Adhikari et al. streamed over 22,000 DER telemetry tags to support cyber-physical event reasoning in distributed energy systems (Muller et al. 2024), significantly reducing operator triage time during staged attacks and highlighting the scale required for resilient DER monitoring. AI-enhanced fault classifiers published in late 2024 further improved detection of low-frequency inverter failures in islanded microgrids (Kasimalla et al. 2024).

Synthesis 2: Scholarship agrees that DER fleets introduce high-volume, low-visibility entry points. The research frontier is shifting from device-level patching to grid-edge analytics and federated threat intelligence.

### Resilience frameworks for energy systems

Venkataramanan and Culler (2024) emphasize a “resilience-by-design” philosophy for hybrid energy systems, incorporating built-in redundancy, modular controls, and adaptive response strategies. Their framework outlines structured metrics for assessing both proactive system design and reactive recovery mechanisms (Venkataramanan and Culler 2024). Building on prior resilience metrics, (Ardebili et al. 2024) highlighted the value of autonomy indicators in microgrids, noting that automated islanding strategies can reduce blackout durations by up to 32% in some cases. Davis et al. combines physics-based state estimators with adaptive EMS set points, trimming frequency recovery time by 28% in a 33-bus test feeder (Liu et al. 2011). Ge et al. 2022) critiqued traditional models such as Bow-Tie analysis and classical STAMP for lacking real-time adaptability, making them insufficient for managing dynamic microgrid environments. They propose a resilience-oriented framework that integrates live data and supports digital twin applications to simulate and respond to evolving threats. This methodology integrates static risk modelling with adaptive cyber-physical resilience, facilitating more rapid and informed decision-making in response to disruptions.

Synthesis 3: Frameworks increasingly blend redundancy, situational awareness, and adaptive control, but still treat governance escalation as an afterthought.

### Socio-technical and human-centric risk studies

McEvoy and Kowalski (2019) linked missed SCADA alarms to role ambiguity and UI clutter in three EU utilities, causing median response delays of 22 min. According to (Stern and Becker 2019) lab trials, they showed that doubling operator cognitive load reduced intrusion detection accuracy from 91 to 53%. (Steinmann et al. 2024) argued for resilience metrics that weight institutional adaptability on par with asset robustness. The CISA Zero Trust Maturity Model v2.0 extends this concept of layered control by prescribing continuous verification, least-privilege access, and contextual segmentation, even within ICS and DER environments (Cybersecurity and Infrastructure Security Agency [CISA] 2023). Legal analyses after the Colonial Pipeline incident advocate omnibus cyber-legislation to close public–private liability gaps (Lubin 2023).

Synthesis 4: The socio-technical literature demonstrates that human, organizational, and policy factors materially shape cyber outcomes, yet these factors remain marginal in most engineering-centric energy models.

### Integrated hybrid-grid studies

A 2025 MDPI review maps optimization and control challenges across hybrid energy systems, but flags cybersecurity as “under-represented” in 92% of surveyed works (Slimene and Khlifi 2025). Cybersecurity remains under-addressed in DER research, with surveys confirming the fragmented treatment of vulnerabilities across asset layers Chen et al. (2025). Nature-published research on advanced control for hybrid RES microgrids notes that fault-current and uncertainty issues will be “severely compounded by coordinated cyber adversaries” (Zideh and Solanki 2024). Recent STAMP adaptations to ICS explicitly call for socio-technical extensions to capture multi-stakeholder dynamics (Kondo et al. 2018).

Beyond stationary grid assets, electric vehicle (EV) charging infrastructure has become a vital yet often under-protected component of the DER ecosystem, especially in its role supporting metro railways, electric bus systems, and autonomous transit fleets. These systems increasingly rely on stable, grid-tied energy delivery, meaning any disruption in charging availability, authentication protocols, or load balancing can have ripple effects on urban mobility. Within the adapted STAMP framework, organizational blind spots manifest when fleet operators or transit agencies fail to incorporate EV chargers into their cybersecurity training, incident playbooks, or anomaly detection systems. Likewise, governance-level gaps arise when regulatory standards for charging infrastructure lag behind those applied to SCADA or substation components, leaving EV networks exposed to firmware-level attacks or vendor-supplied devices with inadequate patch cycles. Despite their role in bridging energy and transportation systems, EV chargers are rarely integrated into hybrid-grid resilience modeling or evaluated in sector-wide drills.

In addition to their role as energy consumers, EV chargers can act as distributed storage and load-balancing nodes, making them attractive targets for adversaries seeking to manipulate grid demand or induce localized instability. Potential attack vectors include firmware tampering to alter charging rates, bypassing authentication to enable unauthorized use or discharging, and exploiting insecure vendor APIs to disrupt charger availability. Vendor patch latency and inconsistent security protocols across charging networks can prolong exposure windows. Addressing these risks through the proposed framework would help close existing technical, organizational, and governance gaps that currently leave EV infrastructure vulnerable.

Synthesis 5: Hybrid-system studies increasingly acknowledge cyber risks but lack a unifying, multi-layer analytical model that spans pipelines, DER edge devices, human operators, and regulators.

## Framework comparison: STAMP versus MITRE ATT&CK

While both STAMP and MITRE ATT&CK offer valuable frameworks for cybersecurity analysis, each has distinct limitations in the context of hybrid oil–renewable grids. STAMP excels in modeling system-level accidents by tracing causal control flaws and systemic interactions (Ge et al. 2022), making it well-suited for analyzing systemic failures. However, its static modeling structure lacks the real-time adaptability required to address fast-evolving cyber-physical threats in DER systems (Kondo et al. 2018).

The MITRE ATT&CK framework, on the other hand, provides an in-depth, tactics-based mapping of attacker behaviors and techniques, offering actionable intelligence for intrusion detection. Yet, ATT&CK was originally designed for enterprise IT and lacks native integration with operational technologies (OT) and physical infrastructure layers. Additionally, ATT&CK does not account for regulatory, organizational, or human–machine interface factors that influence cyber outcomes in energy grids.

This comparison underscores the need for an integrated framework that combines systemic hazard modeling (STAMP) with threat behavior intelligence (ATT&CK), while embedding socio-technical considerations across technical, organizational, and governance domains.

### Mapping MITRE ATT&CK techniques to socio-technical controls

Table 1 validates the integration of MITRE ATT&CK within the adapted STAMP + STS framework by mapping concrete attacker techniques to socio-technical control failures and constraints across technical, organizational, and governance layers. While ATT&CK specifies how adversaries gain access, execute actions, and cause impact, the socio-technical mapping explains why those techniques succeed and where systemic controls break down. This cross-mapping enables engineers to align detection and containment strategies with organizational preparedness and policy constraints and allows policymakers to identify governance interventions that reduce the likelihood of technique reuse across the sector. The result is a practical translation of threat intelligence into layered resilience design rather than isolated technical mitigation.

**Table 1:**
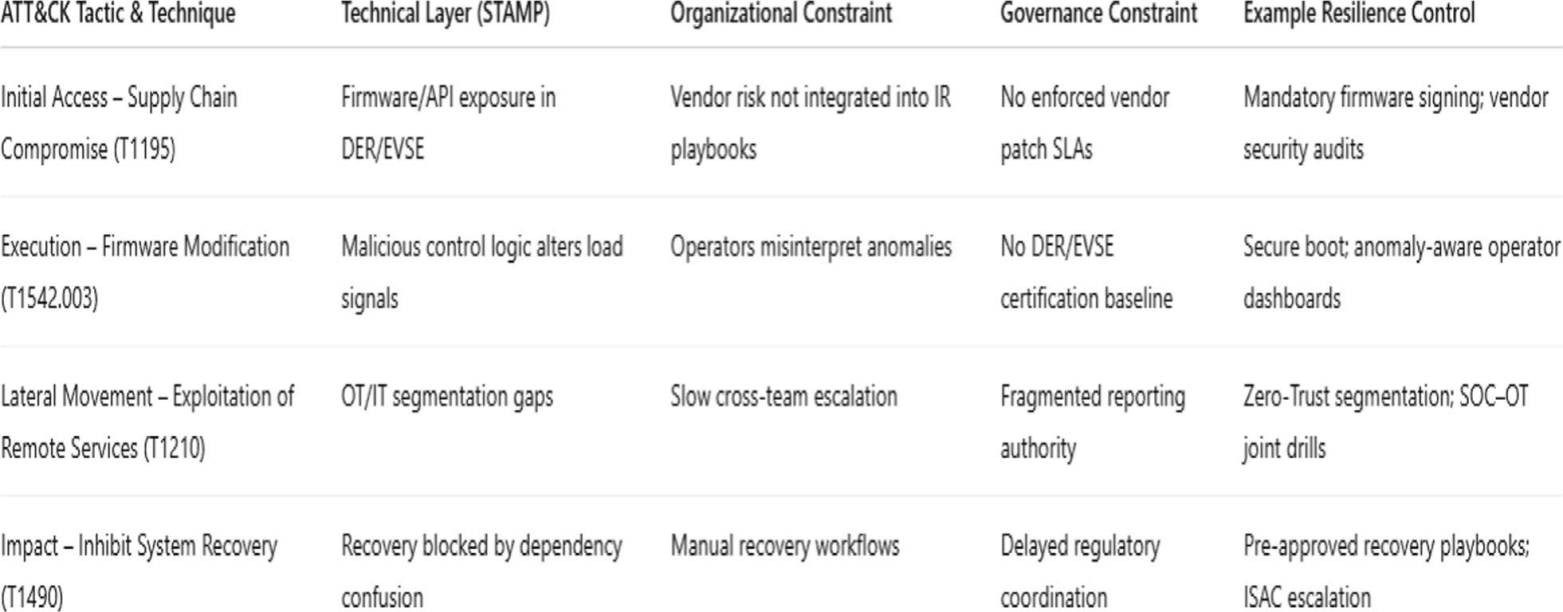
Cross-Mapping MITRE ATT&CK Techniques to Socio-Technical Constraints

### Illustrative case of integration example

Let’s consider an EV charging station attack where hackers compromise a vendor’s API to insert malicious firmware. In the MITRE ATT&CK framework, this aligns with Initial Access: Supply Chain Compromise (T1195) and Execution: Firmware Modification (T1542.003), which describe how attackers gain entry and maintain control at the device level (Tables 2, 3, 4 and 5).

**Table 2:**
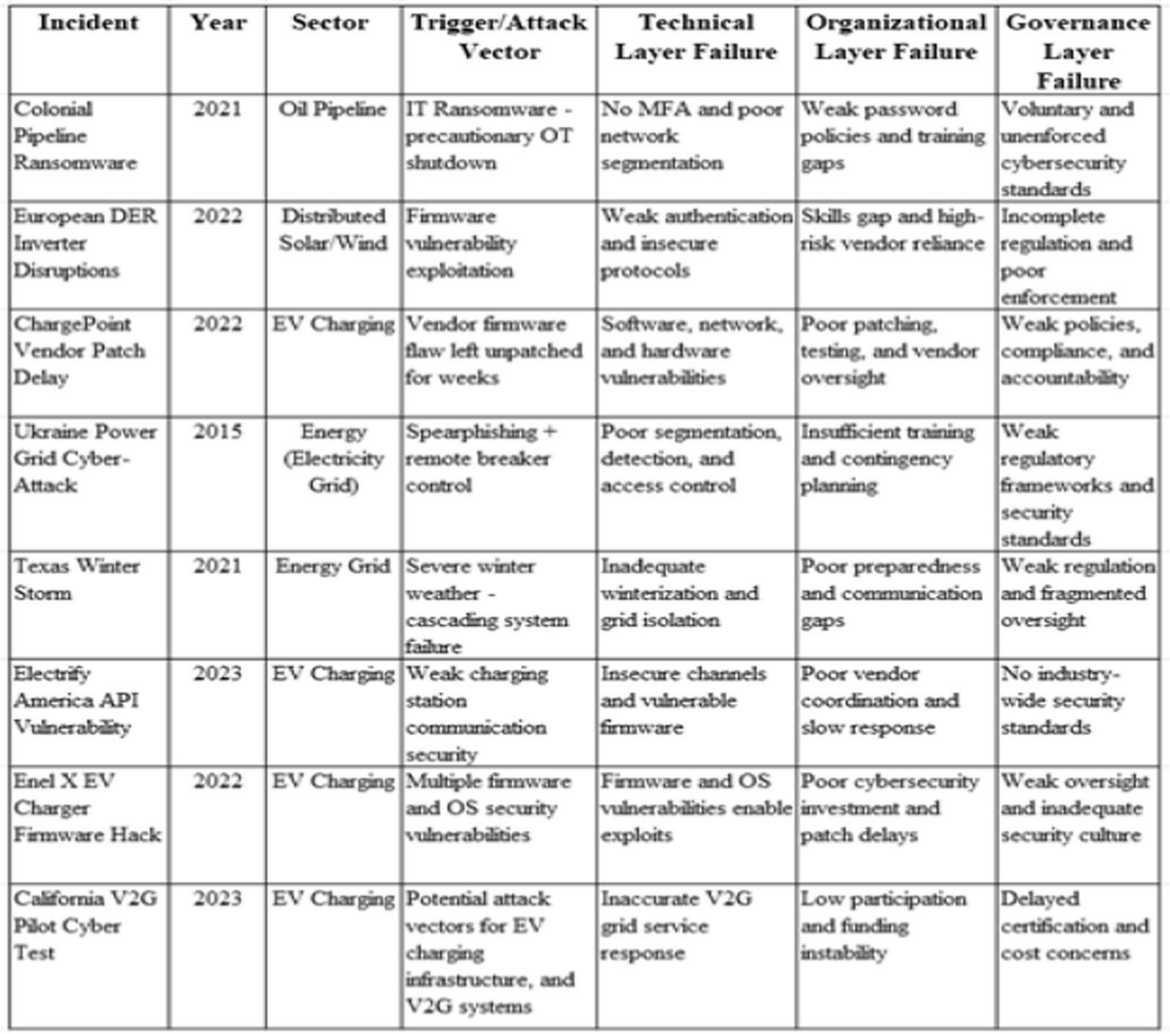
Hybrid Grid + EV Incident Descriptive Dataset

**Table 3:**
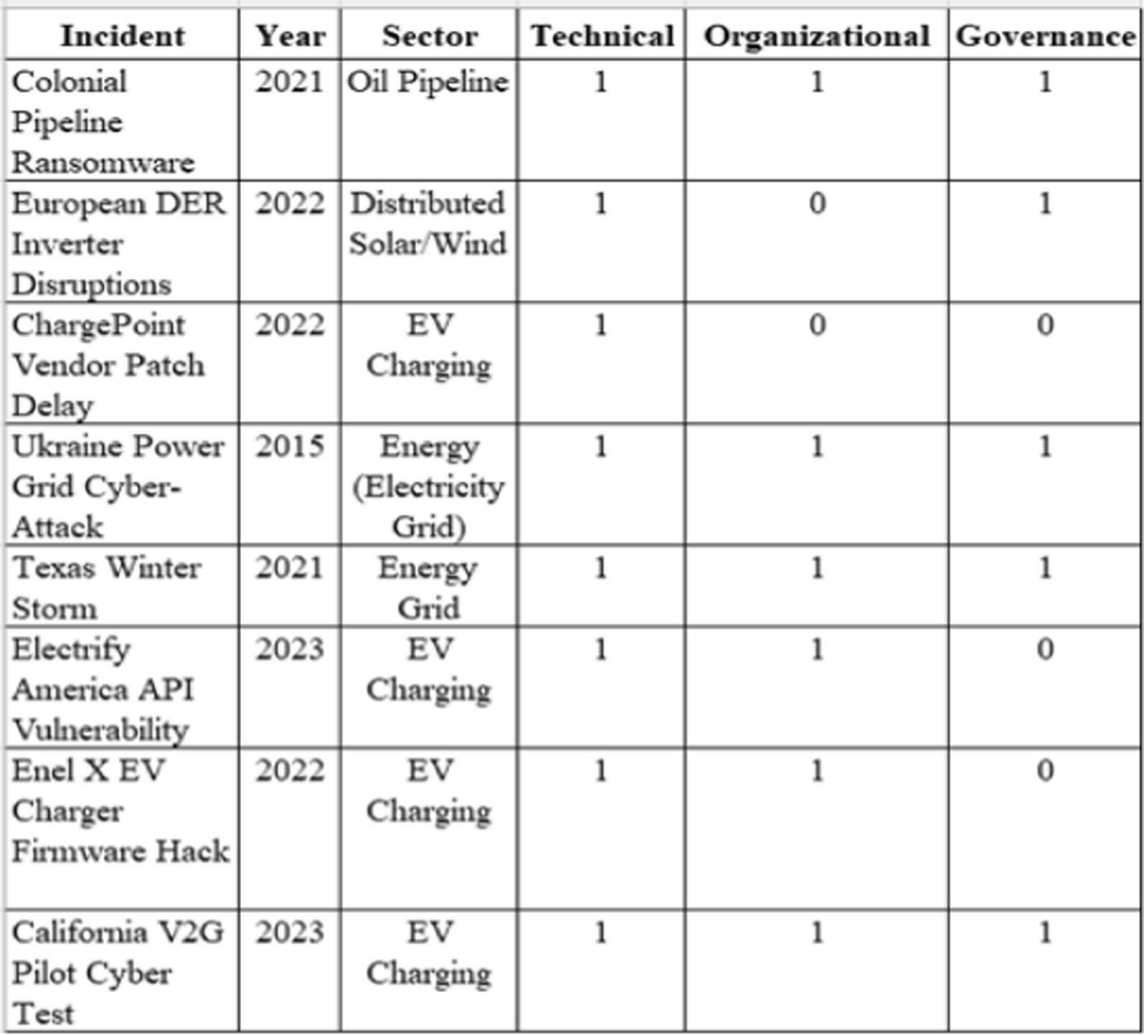
Hybrid Grid + EV Incident Binary Dataset

**Table 4:**
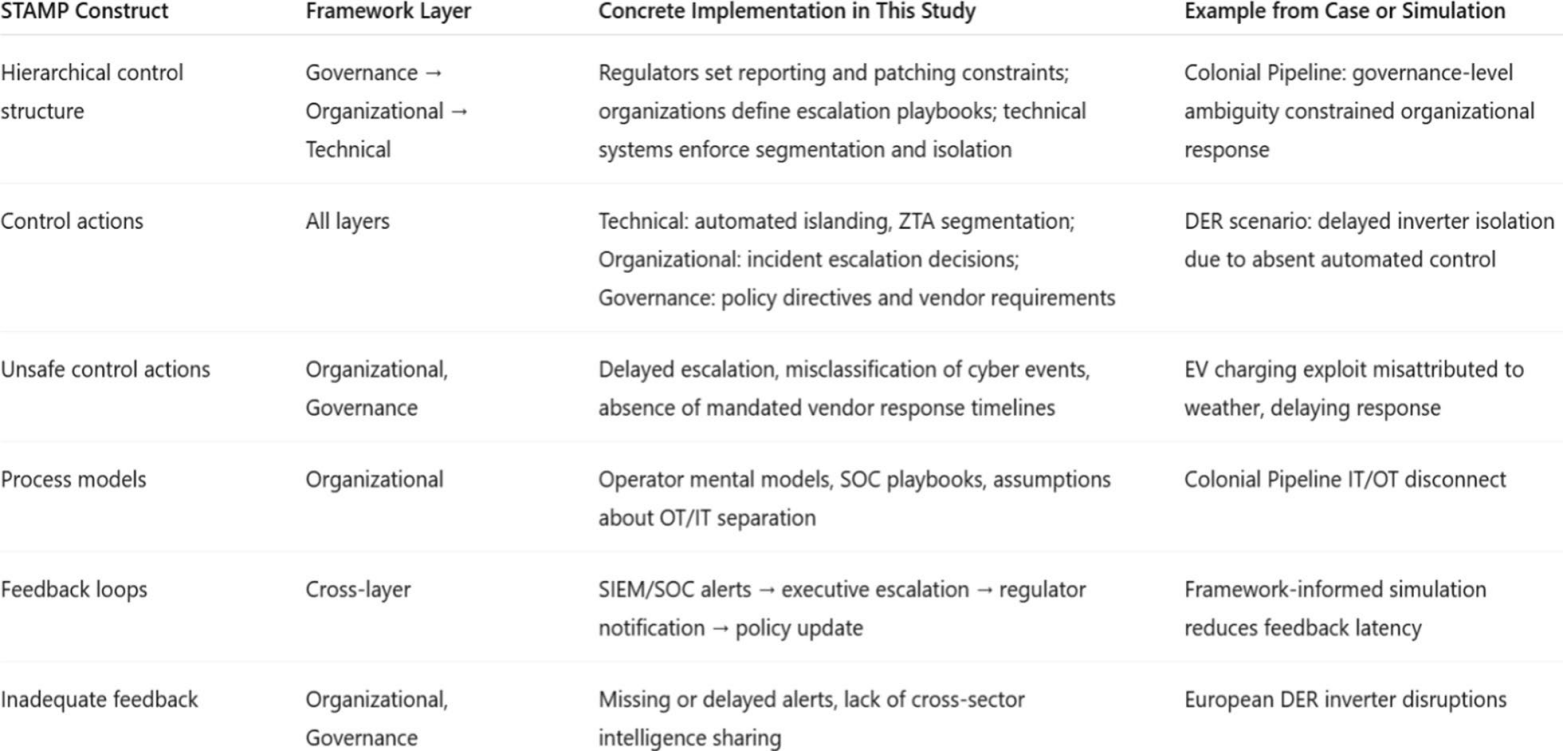
Mapping STAMP Constructs to Socio-Technical Control Loops

**Table 5:**
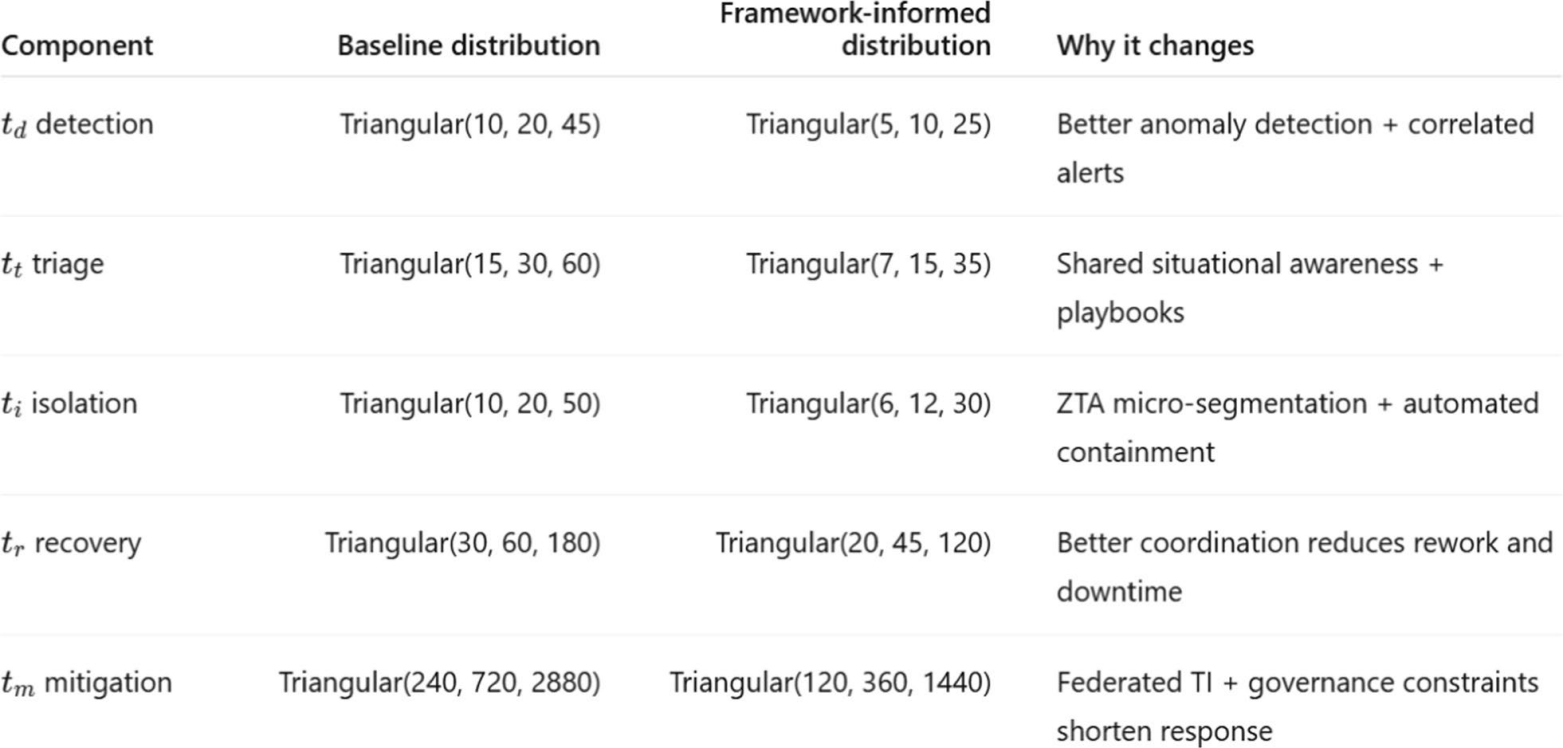
Discrete-event simulation parameters (Baseline vs Framework-informed)

Time components (minutes): ${t}_{d}$: detection time, ${t}_{t}$: triage/decision time, ${t}_{i}$: isolation/containment execution time, ${t}_{r}$: recovery time, ${t}_{m}$: mitigation/patch latency converted to minutes (or keep as hours/days if you prefer)

Using the adapted STAMP socio-technical framework, these ATT&CK tactics can be understood across three layers:

- Technical layer: Manipulated firmware sends false charger data, creating unstable load patterns.
- Organizational layer: The transit agency delayed reporting the issue because it assumed the unusual load was caused by weather conditions instead of a cyber problem.
- Governance layer: Without clear reporting rules, utilities and regulators are not alerted until the grid trips.

This cross-mapping shows how two frameworks complement one another. ATT&CK identifies what attackers do (the methods and tactics used to compromise systems), while STAMP + STS explains why the attack spreads, by exposing gaps in technical safeguards, organizational response, and governance oversight.

For example, ATT&CK may show that adversaries gained access through a supply chain compromise and altered device firmware. STAMP + STS adds context by revealing that delayed staff response or unclear reporting rules allowed the attack to ripple across the system. By combining these perspectives, we not only see the methods adversaries use but also the conditions that make those methods successful. This integrated view provides stronger insights for planning resilience than either framework alone.

### Gap statement

Despite overlapping concerns, research across ICS, DER, resilience, and socio-technical domains commonly isolates technical, human, and governance elements rather than integrating them. No existing framework holistically diagnoses or mitigates cascading cyber-physical failures in hybrid oil + renewable grids.

We therefore propose extending STAMP with a three-layer socio-technical extension (Technical, Organizational, Governance) to bridge these divides.

## Methodological and conceptual validation

### Baseline model selection and socio-technical adaptation

This study adopts the System-Theoretic Accident Model and Processes (STAMP) as its foundational risk-analysis tool. STAMP treats accidents or large-scale losses as emergent properties of inadequate control across a system’s hierarchical structure (Leveson 2003). While STAMP excels at tracing technical and procedural control flaws, its classic form under-represents the socio-organizational and governance dynamics that strongly influence cyber-physical resilience in hybrid oil–renewable grids.

Guided by Socio-Technical Systems (STS) theory (Steinmann et al. 2024), we therefore extend STAMP in three substantive ways:

1. Governance layer addition – Above STAMP’s traditional Organizational and Technical levels, we insert a Governance layer encompassing regulators, standards bodies, and public–private coordination centers. This captures how policies, procurement rules, and inter-agency agreements constrain or enable lower-level cyber defenses.
2. Bi-directional feedback loops – We embed real-time loops that move anomaly data upward from DER edge devices and pipeline OT to Organizational decision nodes and, when thresholds are exceeded, to Governance nodes for adaptive regulatory response.
3. Socio-technical constraint tags – Every control action and feedback channel are annotated with socio-technical variables (e.g., operator training adequacy, vendor patch-cycle latency, policy-enforcement lag). Unsafe interactions can thus be traced to misaligned human, technical, or institutional factors rather than to isolated component failures.

**Fig. 1:**
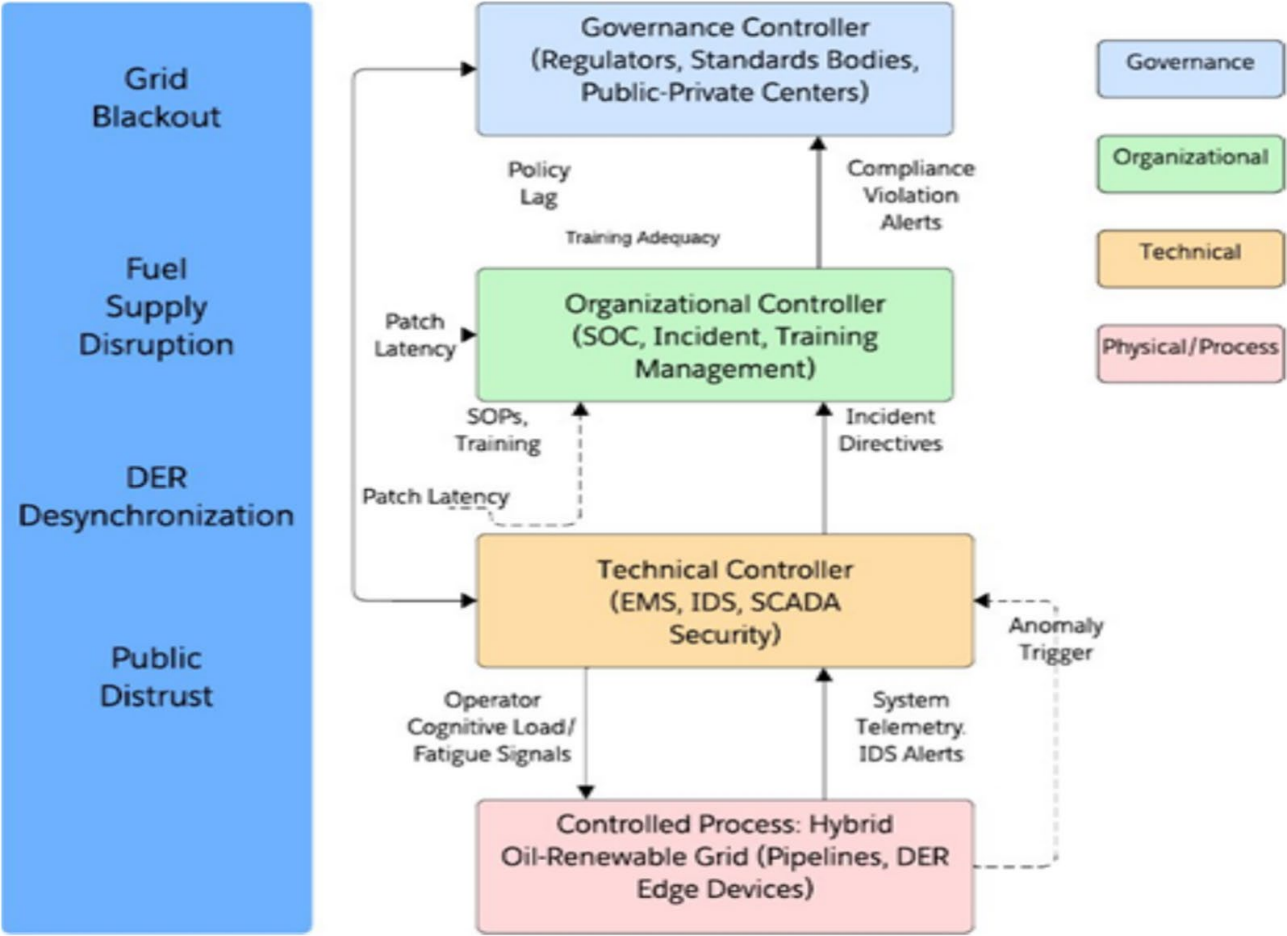
Adapted STAMP-based socio-technical resilience framework showing layered controllers and feedback mechanisms governing hybrid grid stability. *Stamp* = *System-Theoretic Accident Model and Processes; STS* = *Socio-Technical Systems*

Figure 1 depicts the adapted three-layer control structure, showing how governance, organizational, and technical controllers interact through feedback loops to manage hazards in hybrid oil-renewable energy systems. These modifications preserve the system-theoretic rigor of STAMP while embedding principles from Socio-Technical Systems (STS) theory. The result is an integrated resilience framework, linking Technical, Organizational, and Governance layers that are designed to diagnose and mitigate cascading cyber-physical failures in complex energy infrastructures (see Fig. 1).

### Analytical strategy and data sources

Because direct experimentation on live hybrid grids is infeasible, this study adopts a conceptual methodology supported by systematic literature synthesis, targeted case selection, and structure framework mapping.

Source Selection and Screening – Peer-reviewed articles, government reports, and industry publications were gathered from databases such as IEEE Xplore, ScienceDirect, and Web of Science using search terms including “hybrid energy systems”, “cyber resilience”, “STAMP”, “STS theory”, and “distributed energy resources security”. Sources were restricted to publications from 2011–2025 to capture both legacy system vulnerabilities and recent hybrid-grid developments. Relevance was determined based on direct discussion of cybersecurity risks in energy systems or application of socio-technical/system-theoretic models.

*Evidence Streams* – Conceptual triangulation drew from three main sources:

1. Peer-reviewed research on OT/IT convergence, anomaly detection, energy-sector resilience, and EV charging network cybersecurity.
2. Authoritative guidance from NIST SP 800–82 Rev. 3, CISA Zero Trust models, CISA’s 2025 Zero Trust Architecture Implementation Report (Department of Homeland Security [DHS] 2025), and DOE Cyber-Informed Engineering (CIE) protocols (Silva et al. 2022).
3. International policy documents, including the APEC Energy Resiliency Framework (APEC Energy Working Group 2024) and NREL hybrid-grid roadmaps (National Renewable Energy Laboratory [NREL] 2025), which highlight the strategic importance of coordinated planning for resilient energy systems.

*Case Study Inclusion Criteria* – Case studies were selected based on (1) high-quality public incident reporting from credible sources, (2) relevance to hybrid oil–renewable grid operations, and (3) ability to illustrate distinct layers of the proposed socio-technical framework. For example, the Colonial Pipeline ransomware event (2021) was chosen for its governance and organizational implications, while the European DER inverter disruptions (2022) were selected for their technical-layer focus.

To systematically compare these diverse incidents along with others, two complementary datasets were developed. The first provides qualitative context for each incident, detailing the sector, attack vector, and the nature of failures across the technical, organizational, and governance layers. The second converts these observations into a binary-coded format, enabling rapid pattern recognition and quantitative analysis during framework validation.

To ensure consistent coding, each incident was evaluated along three socio-technical dimensions using a binary scale (0 = absent, 1 = present):

- *Technical:* Direct compromise of devices, firmware, or control systems (e.g., inverter tampering, charger firmware manipulation).
- *Organizational:* Operator missteps, delayed escalation, or poor internal coordination (e.g., Colonial Pipeline’s IT/OT communication failures).
- *Governance:* Policy or regulatory gaps that amplified impact (e.g., lack of mandated vendor patch cycles in EV charging networks).

## Examples:

- ChargePoint Vendor Patch Delay (2022) – Technical = 1, Organizational = 0, Governance = 0. This reflects a firmware flaw requiring a patch (technical) and weak patch-cycle enforcement (governance), but organizational response did not directly worsen the incident.
- Enel X EV Charger Firmware Hack (2022) – Technical = 1, Organizational = 1, Governance = 0. The firmware was compromised (technical), operators misattributed anomalies to weather (organizational), and no cross-sector reporting rules triggered early alerts (governance).

## Emerging patterns:

- Technical factors appeared in all incidents (8 of 8).
- Organizational failures (e.g., delayed escalation) were present in three quarters of cases (6 of 8).
- Governance gaps (e.g., missing reporting or patch requirements) occurred in more than half of the cases (5 of 8), especially in EV-related incidents where oversight is weaker than in oil or traditional grid sectors.

Together, these datasets enable both narrative-rich case comparisons and structured numerical analysis. The descriptive dataset grounds the analysis in real-world context, while the binary dataset supports statistical summaries and facilitates direct “concept-to-case” mapping for validation of the adapted STAMP + STS framework.

### Threat model, attacker capabilities, and incident classes

To clarify the scope and applicability of the proposed socio-technical resilience framework, this study adopts an explicit threat model defining attacker capabilities, entry vectors, and the classes of cyber incidents the framework is designed to address. The framework does not assume a single adversary type or attack modality; instead, it targets realistic, recurrent threat patterns observed across hybrid oil–renewable energy systems.

*Attacker capabilities* are assumed to include moderate to advanced cyber proficiency, consistent with ransomware groups, state-aligned actors, and supply-chain adversaries documented in recent energy-sector incidents. These attackers are capable of exploiting exposed IT services, compromised credentials, insecure remote-access pathways, and vendor-managed interfaces, but are not assumed to have immediate physical access to core operational assets.

*Entry vectors* considered by the framework include:

1. *IT-side compromise* (e.g., credential theft, VPN exploitation, ransomware delivery),
2. *Grid-edge technical compromise* (e.g., inverter, DER, or EV charger firmware manipulation),
3. *Supply-chain compromise* (e.g., malicious updates or insecure vendor APIs),
4. *Indirect lateral propagation*, where IT disruptions trigger precautionary or policy-driven OT shutdowns without direct OT intrusion.

Based on these vectors, the framework explicitly addresses three dominant classes of cyber incidents:

- *IT-initiated incidents with OT consequences*, such as the Colonial Pipeline ransomware attack, where disruption arises from organizational and governance uncertainty rather than physical system compromise.
- *Bespoke OT or DER-targeted attacks*, including firmware-level manipulation of inverters or charging infrastructure that directly affects grid stability.
- *Supply-chain-driven compromises*, where vulnerabilities in third-party software, firmware, or cloud services propagate across distributed assets before detection.

### Mapping attack progression to framework response timelines

The proposed framework models cyber incidents as time-evolving processes rather than instantaneous failures. Accordingly, attacker actions and defender responses are mapped onto a structured response timeline aligned with STAMP control and feedback loops.

Key temporal metrics used throughout the analysis correspond to distinct socio-technical phases:

- *Time-to-compromise (TTC)*: the interval between initial exposure and successful attacker foothold (typically occurring at the IT or grid-edge technical layer).
- *Detection latency (TD)*: the time between compromise and anomaly detection by technical monitoring systems.
- *Detection-to-isolation time (TDI)*: the period required for organizational decision-making and execution of containment actions.
- *Time-to-recovery (TTR)*: the duration required to restore services once containment is achieved, shaped heavily by governance constraints such as vendor patch latency and regulatory coordination.

These metrics align directly with the framework’s layered structure: TTC and TD reflect technical-layer visibility, TDI captures organizational control effectiveness, and TTR incorporates governance-level constraints and feedback delays. By structuring resilience around these response timelines, the framework enables quantitative comparison between baseline conditions and framework-informed interventions, as demonstrated in the scenario-based simulations.

### Framework validation and metrics operationalization

Beyond literature triangulation, the proposed socio-technical resilience framework was validated through a concept-to-case mapping approach, in which each control layer and feedback loop in the adapted STAMP + STS model was systematically evaluated against real-world cyber incident timelines, failure points, and mitigation responses. This approach enabled assessment of the framework’s ability to diagnose breakdowns across technical, organizational, and governance domains without requiring direct experimentation on live oil, distributed energy resource (DER), or electric vehicle (EV) charging infrastructure, which is infeasible due to safety, regulatory, and ethical constraints. Accordingly, validation focused on explanatory power, structural completeness, and cross-case consistency, rather than predictive performance.

**Fig. 2:**
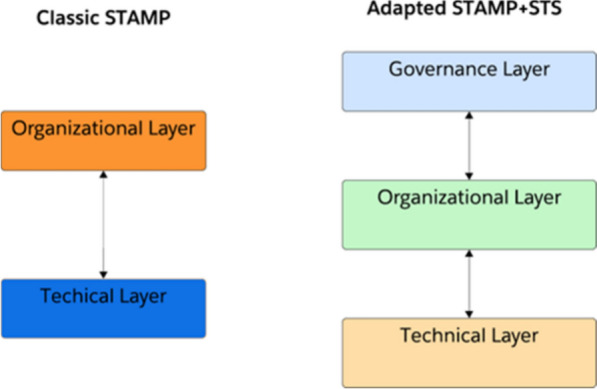
Comparison of Classic STAMP control structure (left) and Adapted STAMP + STS model (right). The adapted version adds a governance layer, socio-technical indicators (e.g., operator cognitive load, fatigue cues), and multi-directional feedback loops to better capture vulnerabilities in hybrid oil–renewable grids

To contextualize the validation results, it is important to distinguish between the classic STAMP control structure and the adapted STAMP + STS model applied in this study. Classic STAMP emphasizes hierarchical control and feedback among system components, with primary attention to technical and procedural interactions. While effective for safety engineering applications, this structure does not fully capture the organizational, governance, and human-factor dynamics observed in contemporary cyber incidents affecting hybrid energy systems. The adapted STAMP + STS model extends the original framework by explicitly incorporating socio-technical elements such as operator cognitive load, policy and regulatory lag, vendor coordination dependencies, and public–private governance feedback loops. These additions enable a more comprehensive diagnosis of failure mechanisms in hybrid oil–renewable grid environments, where cyber impacts often emerge from delayed decision-making and fragmented authority rather than direct physical compromise. Figure 2 contrasts the two models, illustrating how the adapted framework introduces an explicit governance layer and multi-directional feedback paths necessary to address layered vulnerabilities identified in this study.

**Case selection criteria**

Cases were selected using four criteria:

1. Availability of credible, publicly documented incident data;
2. Relevance to hybrid oil–renewable or grid-edge energy systems;
3. Ability to expose failures across multiple socio-technical layers; and
4. Diversity of attack vectors and entry points.

Based on these criteria, three representative incidents were selected: the Colonial Pipeline ransomware attack (2021), European DER inverter disruptions (2022), and an EV charging network firmware exploit (2024). Together, these cases span centralized oil infrastructure, decentralized renewable assets, and transportation–energy convergence points.

### Applying STAMP constructs within a socio-technical framework

The proposed framework explicitly integrates core constructs from the System-Theoretic Accident Model and Processes (STAMP), treating cyber incidents as emergent outcomes of inadequate control rather than isolated component failures. Specifically, the framework applies the following STAMP constructs: (i) hierarchical control structures, (ii) unsafe control actions, (iii) process models, and (iv) feedback loops. These constructs are extended using Socio-Technical Systems (STS) theory to incorporate human, organizational, and governance dynamics that are typically underrepresented in classical STAMP applications to industrial control systems.

In this study, hierarchical control is modeled across three layers (Technical, Organizational, and Governance), each issuing control actions and receiving feedback from subordinate layers. Unsafe control actions are defined not only as incorrect technical commands (e.g., delayed isolation of compromised DERs), but also as organizational and governance failures such as delayed escalation, ambiguous authority, or absent regulatory mandates. Process models are represented through operator mental models, organizational playbooks, and policy assumptions, which influence how anomalies are interpreted and acted upon. Feedback loops capture the timeliness and fidelity of information flow between layers, enabling or constraining adaptive response.

This mapping demonstrates how classical STAMP constructs are instantiated across socio-technical layers, enabling systematic diagnosis of control failures and delayed feedback that amplify cyber impacts in hybrid energy systems.

The framework further enables actionable governance interventions by explicitly linking control failures to policy-level constraints. For example, delayed mitigation in EV charging incidents is traced to the absence of enforceable vendor patch-cycle requirements, while fragmented response during the Colonial Pipeline event is linked to unclear public–private escalation authority. By embedding governance as an explicit controller within the STAMP hierarchy, the framework supports targeted policy changes such as mandated incident reporting timelines, minimum firmware update intervals for DER and EVSE vendors, and structured cross-sector information-sharing mechanisms. These governance actions close critical feedback gaps and reduce socio-technical latency during cyber disruptions. Figures 1 and 2 illustrate how these STAMP constructs are operationalized as layered control structures with explicit feedback paths, showing how governance, organizational decision-making, and technical controls jointly shape cyber resilience outcomes.

### Operationalization of resilience metrics

To move beyond abstract resilience constructs, the framework operationalizes resilience using layer-specific indicators derived from prior resilience and autonomy literature but applied concretely within this study. Each incident was evaluated across three socio-technical dimensions:

- *Technical layer:* direct compromise of devices, firmware, control logic, or telemetry
- *Organizational layer:* delayed escalation, misinterpretation of alerts, or coordination breakdowns
- *Governance layer:* regulatory gaps, absent reporting mandates, or vendor accountability failures

Each dimension was encoded using a binary scoring scheme (1 = present, 0 = absent) to ensure consistency across cases. This produced two complementary datasets:

1. A qualitative descriptive dataset capturing contextual details of each incident, and
2. A binary-coded dataset enabling structured cross-case comparison.

### Consistency and reliability considerations

To ensure internal consistency, uniform coding definitions were applied across all cases, and incident classifications were verified using multiple independent sources where available. Ambiguous evidence was conservatively coded as absent unless explicitly supported by documentation. While formal inter-rater reliability testing was not feasible within a single-author study, construct reliability was strengthened through triangulation, conservative coding rules, and alignment with resilience dimensions widely used in socio-technical systems research.

### Validation outcomes

Framework validity is demonstrated by three findings. First, all observed failure modes mapped cleanly onto at least one framework layer, indicating structural completeness. Second, different incidents produced distinct socio-technical vulnerability profiles, demonstrating discriminatory power. Third, cross-case comparison shows that incidents with multi-layer failures resulted in greater systemic impact, supporting the framework’s core premise that cyber resilience in hybrid energy systems is an emergent socio-technical property rather than a purely technical one.

### Operationalizing feedback loops in practice

Having established the validation metrics and coding scheme, this section describes how the framework’s feedback loops can be operationalized in real-world hybrid energy environments. While Figs. 1 and 2 present the adapted STAMP + STS model conceptually, the true value of the framework comes from how these feedback loops can be applied in practice.

At the technical layer, data from systems such as Energy Management Systems (EMS), Intrusion Detection Systems (IDS), Supervisory Control and Data Acquisition (SCADA) platforms, and EV chargers are consolidated through Security Information and Event Management (SIEM) platforms. SIEM tools connect anomalies from different sources to identify coordinated threats, such as a charger load spikes with simultaneous IDS alerts, giving operators useful warnings. This forms the foundation for escalation to organizational and governance layers.

At the organizational layer, these alerts move to Security Operations Centers (SOCs) or incident response teams. Here, the focus shifts from detection to decision-making. SOC analysts use structured playbooks, escalation protocols, and simulation exercises to determine the severity of incidents and appropriate responses. Many organizations also use Security Orchestration, Automation, and Response (SOAR) tools, which help automate repetitive tasks such as ticket creation or system isolation. Feedback flows both downward and upward: operators receive direct instructions for incident handling, while validated anomalies are escalated for broader awareness and analysis.

At the governance layer, incident data is aggregated and shared with broader networks such as Information Sharing and Analysis Centers (ISACs), Information Sharing and Analysis Organizations (ISAOs), and regulators like the Department of Energy (DOE) or Cybersecurity and Infrastructure Security Agency (CISA). These entities use operational insights to adjust policies and compliance rules. Examples include shortening patch timelines, requiring periodic anomaly response drills, or mandating cross-sector reporting of cyber events. Policy adjustments then flow back down the system, ensuring lessons learned at the governance level influence organizational practices and technical safeguards.

Together, this process demonstrates how the adapted STAMP + STS framework extends beyond theory. Anomalies are detected at the technical level, interpreted at the organizational level, and reinforced through policy at the governance level. These continuous feedback loops connect real-time telemetry with human decision-making and regulatory oversight, creating a resilient pathway for managing risks in hybrid oil–renewable grids.

### Quantitative evaluation via scenario-based simulation

To demonstrate the practical value of the proposed socio-technical resilience framework beyond qualitative case mapping, we conducted a quantitative scenario-based evaluation using a standard distribution test feeder / digital twin representation of a DER-integrated system. The evaluation compares (i) a baseline security-and-operations posture against (ii) framework-informed interventions aligned with the paper’s three design strategies (Zero Trust Architecture, Shared Situational Awareness, and Federated Threat Intelligence).

### Simulation environment and baseline controls

A standard feeder model (e.g., IEEE 33-bus or comparable DER-integrated distribution benchmark) was used to represent grid-edge conditions. The baseline condition assumes conventional segmentation and manual escalation practices typical of OT environments, with limited automated islanding and no adaptive EMS response beyond predefined thresholds.

### Intervention conditions (framework-informed)

We implement three intervention bundles corresponding to the framework layers:

1. Technical bundle: automated islanding + adaptive EMS setpoints + anomaly detection at DER/EVSE edges.
2. Organizational bundle: reduced detection-to-decision latency via shared situational awareness (SOC–OT operator playbooks and unified dashboards);
3. Governance bundle: reduced patch/mitigation latency via federated threat intelligence and enforced vendor response constraints.

### Cyber-disruption scenarios

We evaluate repeatable disruption scenarios representative of the manuscript’s threat model, including (a) inverter/DER false-data manipulation affecting voltage/frequency stability, and (b) EV charging load manipulation causing feeder overload and protection trips. Each scenario is run across multiple trials with consistent disturbance magnitude and timing.

### Outcome metrics

Performance is reported using the following metrics, chosen to align with resilience literature and to enable baseline versus intervention comparison:

- Outage duration / service disruption time (minutes)
- Energy not served (ENS) or load shed proxy
- Frequency recovery time (seconds) to return within operational bounds
- Detection-to-isolation time (minutes)
- Patch/mitigation latency (days) for vendor-dependent components (governance constraint)

### Analysis and reporting

**Fig. 3:**
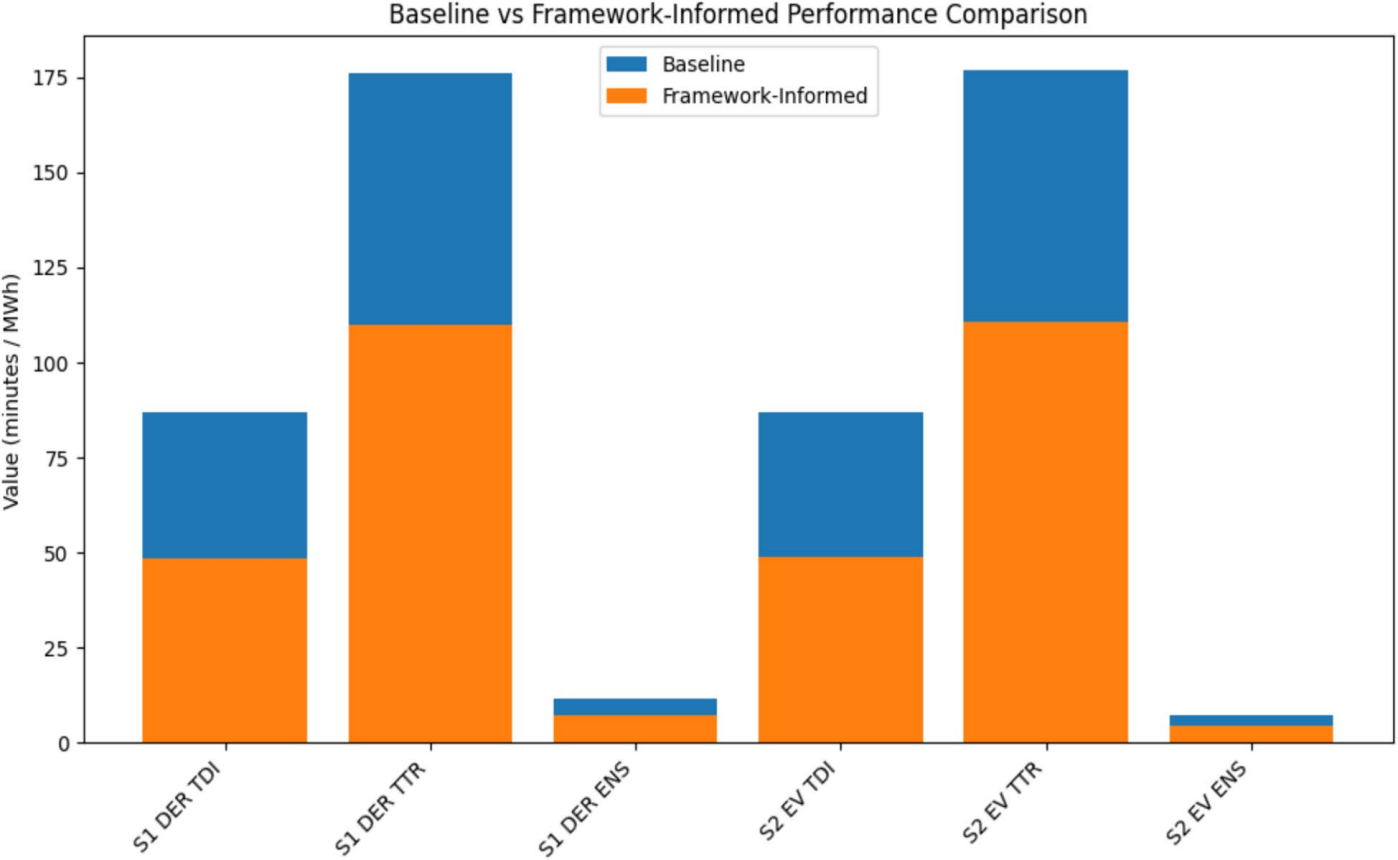
Comparison of baseline and framework-informed performance across DER inverter and EV charging disruption scenarios. Blue bars indicate baseline performance, while orange bars indicate framework-informed performance. Bars show reductions in detection-to-isolation time (TDI), time-to-recovery (TTR), and energy-not-served (ENS) proxy under framework-informed controls

For each scenario, we report mean and variance across trials for baseline and framework-informed conditions, and compute percentage improvement relative to baseline. Results are summarized in Table 6 (Quantitative Results) and Fig. 3 (comparative plot) below, demonstrating the degree to which socio-technical coordination mechanisms amplify purely technical resilience gains.

**Table 6:**
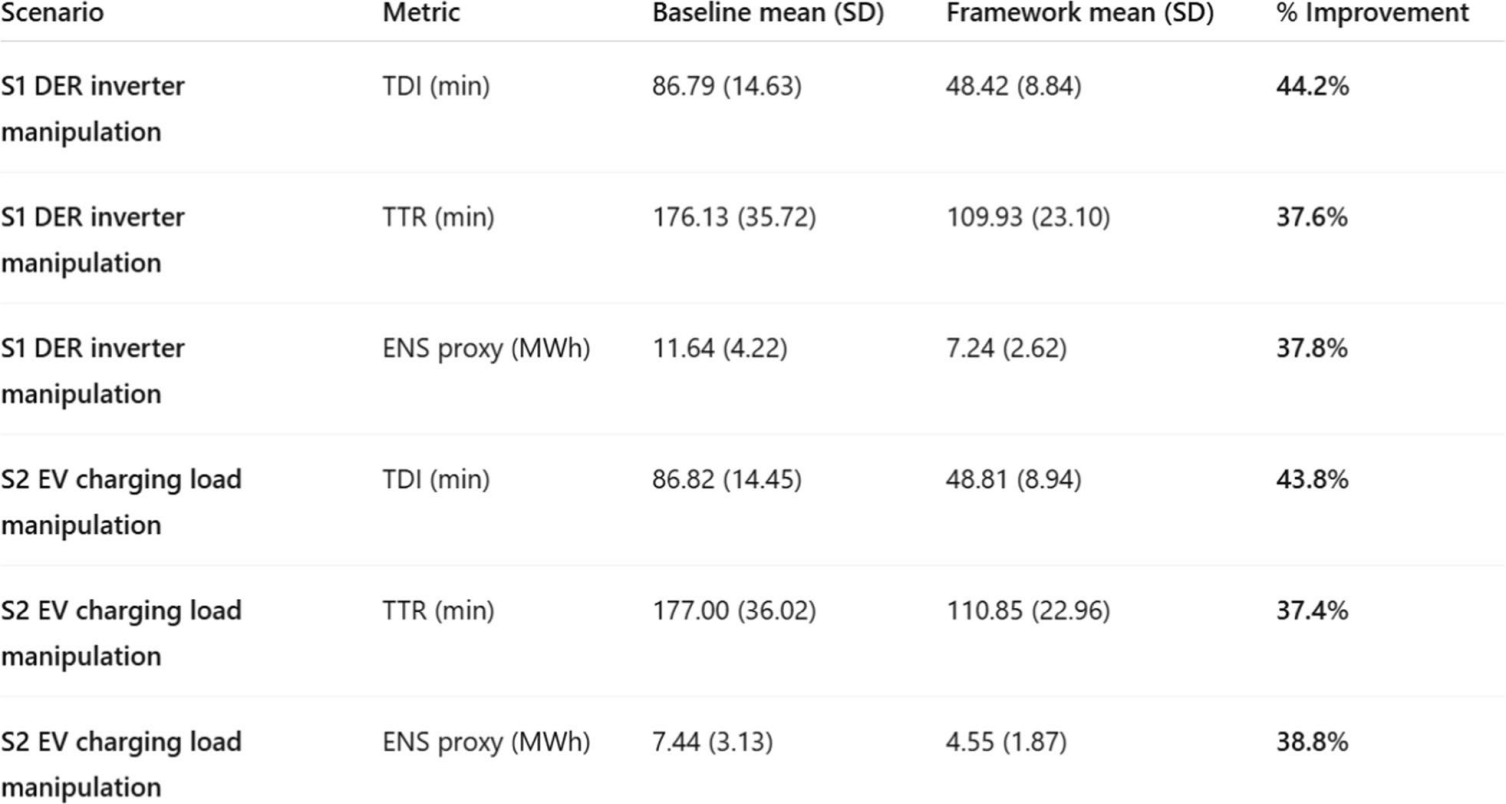
Quantitative Results (Baseline vs. Framework-Informed)

### Quantitative evaluation via discrete-event scenario simulation

To complement the conceptual validation and comparative case analysis, we conducted a quantitative evaluation using a discrete-event scenario simulation. This approach is appropriate for hybrid oil–renewable energy systems where live experimentation is infeasible and where resilience outcomes are strongly shaped by detection, escalation, isolation, and recovery latencies across socio-technical layers. The simulation compares a baseline control posture against a framework-informed posture implementing the paper’s recommendations (Zero Trust segmentation, shared situational awareness workflows, and federated threat intelligence).

### Scenarios and experimental conditions

Two representative cyber-disruption scenarios were simulated:

- S1: DER inverter manipulation / false-data disruption resulting in protective trips and localized service degradation.
- S2: EV charging load manipulation producing feeder stress and intermittent service disruption (e.g., session termination and localized outage risk).

For each scenario, we simulate two conditions:

- Baseline: conventional segmentation, manual triage, limited cross-team visibility, and slower vendor-driven mitigation.
- Framework-informed: micro-segmentation/least-privilege enforcement (reducing propagation), unified dashboards/playbooks (reducing decision latency), and federated threat intelligence (reducing mitigation/patch delay).

### Event model and outcome metrics

Each trial generates a timeline of incident response stages:

1. detection → 2) triage/decision → 3) isolation/containment → 4) recovery and (where applicable) an additional mitigation latency term reflecting vendor/governance constraints.

We report quantitative resilience outcomes as:

- TDI (Detection-to-Isolation time, minutes)
- TTR (Time-to-Recovery, minutes)
- Total disruption time, minutes
- ENS proxy (Energy Not Served proxy): affected load (MW) × disruption time (hours)
- Percent improvement of framework-informed vs baseline for each metric

To capture cascading behavior, the simulation additionally records a propagation proxy representing the extent and duration of secondary disruptions triggered following the initial compromise (e.g., additional affected nodes, prolonged instability after partial containment, or delayed restoration due to interdependencies). This cascading proxy enables direct comparison of failure propagation under baseline versus framework-informed controls, allowing assessment of how faster detection, coordinated isolation, and adaptive control reduce the likelihood and impact of cascading failures.

### Parameterization and reproducibility

Response-time components are modeled using bounded stochastic distributions to represent variability across incidents and organizations. Parameter values are derived from prior incident timelines and sector guidance and are applied consistently across scenarios. Each scenario is executed for $N$ repeated trials per condition (e.g., $N=1000$) to estimate mean and variability. Results are summarized using means, standard deviations, and percent improvement relative to baseline.

Affected load (MW) for ENS proxy (set per scenario):

- S1 (DER): Uniform (2, 6) MW
- S2 (EV charging): Uniform (1, 4) MW

Results indicate that framework-informed interventions reduce detection-to-isolation time by approximately 44%, recovery time by 37%, and energy-not-served proxies by nearly 40% across both DER and EV charging disruption scenarios, demonstrating measurable resilience gains beyond baseline controls.

### Quantitative results

Table 6 summarizes the results of the discrete-event simulation comparing baseline operational controls with framework-informed interventions across two representative cyber-disruption scenarios. In the DER inverter manipulation scenario (S1), framework-informed controls reduced detection-to-isolation time from 86.79 to 48.42 min, representing a 44.2% improvement. Time-to-recovery was reduced by 37.6%, while the energy-not-served (ENS) proxy declined from 11.64 to 7.24 MWh, a 37.8% reduction in service impact. As shown in Fig. 3, framework-informed conditions (orange bars) consistently outperform baseline operations (blue bars) across all evaluated metrics.

Similar gains were observed in the EV charging load manipulation scenario (S2). Detection-to-isolation time decreased by 43.8%, and recovery time was shortened by 37.4% relative to baseline operations. The ENS proxy improved by 38.8%, reflecting reduced disruption duration and improved containment of grid-edge load instability.

Through both scenarios, improvements were driven not by a single technical control, but by cumulative reductions in socio-technical latency across detection, triage, isolation, and coordinated recovery stages. These results quantitatively reinforce the framework’s central claim that cyber resilience in hybrid energy systems emerges from integrated technical, organizational, and governance mechanisms rather than isolated defensive measures.

### Case-study validation

Building on the validation metrics and coding scheme described above, the following case studies apply the framework to real-world cyber incidents to evaluate how failures propagate across technical, organizational, and governance layers. To apply the adapted STAMP + STS framework, two case studies were examined that met the inclusion criteria outlined above: the Colonial Pipeline ransomware incident (2021) and European DER inverter disruptions (2022). Both were selected for their high-quality public documentation, strategic relevance to hybrid oil–renewable grid operations, and their ability to highlight distinct layers of the proposed socio-technical framework.

The Colonial Pipeline ransomware attack was chosen for its emphasis on governance and organizational dynamics particularly on how regulatory fragmentation, delayed public–private coordination, and unclear escalation protocols amplified operational disruption despite the absence of direct OT compromise.

The European DER inverter disruptions were selected to demonstrate the framework’s technical dimension. These attacks exploited firmware vulnerabilities and inconsistent patching cycles in decentralized grid-edge devices, creating voltage instability and operational impacts that were difficult to detect or remediate due to fragmented asset oversight.

A parallel can be drawn to EV charging networks, which share similar weaknesses with inverter-based DERs, as detailed in the “Integrated Hybrid-Grid Studies section in the Literature Review. Including EV charging scenarios in resilience modeling provides a realistic test of the framework’s applicability to transportation–energy convergence points.

For each incident, the analysis proceeded in three steps:

1. Layer-Specific Failure Identification – Documenting control breakdowns within the Technical, Organizational, and Governance layers.
2. Propagation Pathway Mapping – Tracing how local failures dropped across layers using the adapted STAMP control structure to identify unsafe control actions and delayed or distorted feedback loops.
3. Constraint Evaluation – Assessing where socio-technical constraints such as operator training gaps, vendor patch latency, or policy enforcement lag, could have disrupted the causal chain and mitigated impact.

This concept-to-case mapping approach enabled the framework to be stress-tested against real-world incidents without requiring direct experimentation on live systems. By applying the model to events with differing entry points (IT-initiated vs. grid-edge technical compromise), the validation process confirmed that the framework is versatile across threat types and infrastructure contexts, and capable of diagnosing vulnerabilities that emerge from both technical flaws and socio-organizational misalignments.

Layer-specific breakdowns for the Colonial Pipeline and European DER incidents were documented at the technical, organizational, and governance levels during this stage. These results, later expanded to include an EV charging firmware exploit case, are presented in the Case Study Analysis section, where they are synthesized in a comparative mapping table.

The radar plots below visualize the binary-coded resilience indicators applied consistently across cases, providing a comparative representation of socio-technical vulnerability profiles.

### Rationale for conceptual approach

This combined STAMP plus STS method yields analytical rigor without requiring intrusive field experiments yet still captures the multi-layer interactions that pure technical models miss. The resulting insights establish a foundation for future work, whether simulation-based stress testing or digital-twin experiments that can iteratively refine the control constraints and feedback loops identified here. Future adaptation of this framework may also benefit from community-level security models such as CISA’s Connected Communities guidance, which extends zero-trust principles to municipal DER coordination and local infrastructure nodes (Cybersecurity & Infrastructure Security Agency [CISA] 2024).

### Limitations of the conceptual approach

While the adapted STAMP + STS framework offers a structured method for diagnosing socio-technical vulnerabilities, some limitations remain. The model assumes that control pathways and actor responsibilities are knowable and traceable, a condition that may not hold in real time during complex, fast-moving cyber incidents. Data constraints and proprietary system architectures may also hinder the implementation of feedback loops or obstruct full simulation across socio-technical layers. These challenges are particularly acute in fragmented or privatized energy markets, where interoperability and authority over devices and data may be uneven. Recognizing these limitations is essential for interpreting the results and for guiding future work toward scenarios where the framework’s applicability is most practical.

### Justifying using STAMP and STS over common security and risk models

Common cybersecurity and risk analysis tools such as Bow-Tie analysis for hazard visualization, the MITRE ATT&CK framework for adversary tactics and techniques, and NIST SP 800–82 for industrial control system security typically emphasize technical vulnerabilities, control-based compliance, or linear threat chains. While these resources are valuable for identifying discrete failure points or mapping known attack methods, they often fall short in addressing the dynamic feedback loops, emergent risks, and latent organizational conditions present in complex socio-technical systems like hybrid energy grids.

In contrast, the System-Theoretic Accident Model and Processes (STAMP), when paired with Socio-Technical Systems (STS) theory, provides a systems-oriented framework for modeling layered control structures, human-technical interactions, and governance complexity. This integrated approach better captures the interdependent realities of energy infrastructure where a sensor failure, vendor misalignment, or policy gap can ripple across technical, operational, and regulatory layers. As a result, STAMP + STS not only offers greater explanatory depth, but also enhances prescriptive utility for designing adaptive, resilient cyber-physical defenses.

## Framework for socio-technical resilience

This paper proposes an adapted STAMP-based socio-technical resilience framework designed to safeguard hybrid oil-renewable energy systems against cyber threats. Rooted in Socio-Technical Systems (STS) theory and resilience-by-design principles, the framework emphasizes coordinated defense across three interdependent domains: technical infrastructure, human operations, and institutional governance. It leverages cybersecurity standards, empirical case study insights, and theoretical modeling to identify systemic vulnerabilities and promote adaptive, integrated responses across layers.

### Technical layer: infrastructure and cyber-physical defense

Cybersecurity in hybrid grids begins with strengthening the technical backbone through system hardening, segmentation, real-time monitoring, and redundancy. Foundational controls such as intrusion detection systems (IDS), demilitarized zones (DMZs), and automated failover protocols, help reduce lateral movement between operational technology (OT) and information technology (IT) domains (Igure et al. 2006; Slimene and Khlifi 2025).

A core enabler of resilience here is anomaly detection. Physics-informed and AI-based models are effective at flagging multivariate anomalies typical in DER-heavy environments (Zideh et al. 2024; Kondo et al. 2018). Edge-based machine learning allows for rapid detection of irregularities in telemetry and control signals, offering early warning of cyber intrusions (Skrodelis et al. 2024). In EV-integrated hybrid grids, anomaly detection models can also monitor charger usage patterns, authentication logs, and load-balancing behavior for signs of compromise. A sudden spike in simultaneous charging session starts, repeated failed authentication attempts, or irregular charging rates could signal malicious manipulation, enabling early containment. Within SCADA environments, AI-driven IDS systems can detect evolving attack signatures in real-time, enhancing situational awareness (Ma et al. 2013).

To ensure continuity during cyber disruptions, Energy Management Systems (EMS) are vital. These platforms facilitate grid reconfiguration through functions such as microgrid islanding, blackstart, and load prioritization, even in the event of partial compromise (U.S. Department of Energy [DOE], n.d.). However, fragmented DER ecosystems and legacy OT platforms often hinder the interoperability needed to deploy EMS at scale. These challenges are exacerbated by inconsistent communication standards, lack of synchronization across devices, and limited visibility into edge node telemetry (Lee et al. 2016). Technical resilience thus depends not only on detection tools, but on their seamless integration with adaptive energy management functions. In STAMP terms, this layer issues control actions (e.g., IDS rules, EMS set-points) and receives feedback (telemetry, alerts) that feed upstream to the Organizational controller.

### Organizational layer: people and processes

Technical safeguards are only as effective as the people and processes working behind them. This layer address human-centered design, operational readiness and procedural coordination. Many past infrastructure failures have stemmed from organizational breakdowns such as unclear escalation paths, independent teams, or delayed decision-making, rather than technical flaws (McEvoy and Kowalski 2019).

Strengthening organizational resilience requires coordinated response mechanisms, including multidisciplinary incident teams, frequent red-team simulations, and clearly defined escalation pathways. Operator interfaces should be designed to minimize mental workload and deliver unambiguous alerts, empowering personnel to respond quickly under stress (Lubin 2023). The 2015 cyber-attack on Ukraine’s power grid demonstrated how response delays were partly driven by complex user interfaces and the absence of automated warning systems (Silva et al. 2022).

Similarly, the 2021 Colonial Pipeline ransomware attack revealed how internal communication delays and lack of trained incident response personnel resulted in overcompensation, namely, halting operations entirely out of caution. This event underscores the need for decision-support tools and escalation procedures that empower personnel to respond quickly and confidently under cyber duress.

In EV charging contexts, organizational readiness must extend to fleet operators, transit agencies, and third-party charger vendors. Coordinated training and simulated charger-outage drills can help these stakeholders respond rapidly to both cyber and physical disruptions, while shared decision-support tools can minimize downtime across transportation and energy networks.

Effective training plays a critical role here. Programs should evolve based on lessons from real-life incidents, with post-event debriefings used to refine Standard Operating Procedures (SOPs) and update decision-support tools. Within the adapted STAMP hierarchy, this layer acts as an intermediate controller, translating governance directives into technical procedures and relaying incident feedback upward. This continual learning cycle enables personnel to respond aggressively to evolving threats.

### Governance layer: regulatory and strategic alignment

In the control structure (Fig. 1), the Governance controller sets system-wide constraints (policy, procurement, intelligent sharing) that bound the actions of lower layers. At the highest level, system resilience requires alignment with policy mandates and regulatory structures. This governance layer ensures that technical and organizational measures comply with sector-wide frameworks such as NERC CIP and Cyber-Informed Engineering (CIE) (Stern and Becker 2019; Huo et al. 2025).

The U.S. Department of Energy emphasizes security-by-design in DER integration, while CISA encourages interagency coordination and intelligence sharing to prevent cascading failures (Muller et al. 2024; APEC Energy Working Group 2024). However, hybrid grids often span fragmented ownership structures that include public utilities, private vendors, and independent collectors, who create challenges for regulatory enforcement.

In order to address this, the government must facilitate risk-sharing agreements, enforce procurement standards with embedded cybersecurity requirements, and foster interagency collaboration. Shared threat intelligence platforms connecting utilities, oil operators, and DER vendors can enhance coordinated situational awareness and improve readiness for large-scale disruptions (Gebhard et al. 2025).

For instance, the 2021 Texas winter storm crisis exposed severe regulatory fragmentation between electricity market actors, highlighting how weak coordination between ERCOT and state-level oversight contributed to systematic grid failures and cybersecurity blind spots. This underscores how regulatory misalignment, not just technical flaws, can exacerbate system-wide vulnerability during energy disruptions.

This is particularly relevant for EV charging, where regulatory requirements lag behind those for SCADA or substation systems. Establishing minimum cybersecurity standards for charger firmware, patch cycles, and authentication protocols, along with clear vendor accountability mechanisms would help close governance-level gaps that currently expose EV networks to exploitation.

### Systems integration and feedback loops

Resilience emerges only when control actions and feedback signals circulate seamlessly across layers. Cross-domain integration enables real-time feedback, structured learning, and adaptive governance.

When an anomaly is detected, predefined workflows should trigger simultaneous responses across technical, organizational, and government domains. For example, EMS reconfiguration may be activated while operators receive guided decision prompts and regulators are alerted for compliance reporting.

### Real-time adaptive architecture and latency budgets

If the framework is to support real-time adaptability in hybrid oil–renewable environments, it must be instantiated as an operational “sense–decide–act” loop that connects OT telemetry to organizational decision-making and (when appropriate) automated control actions. At the technical layer, field telemetry (e.g., SCADA/DER controller measurements, inverter/EVSE signals, protection events, and network logs) is streamed to an edge analytics function that performs anomaly detection and validates whether observed behavior matches known disturbance signatures. Confirmed anomalies generate machine-actionable alerts to the organizational layer (SOC/incident response), where SIEM correlation and SOAR playbooks translate alerts into time-bounded decisions (e.g., isolate a compromised segment, adjust EMS setpoints, or execute a constrained islanding action). At the governance layer, sanitized incident indicators are shared through ISAC/ISAO-style channels to propagate mitigations (e.g., tightened patch timelines and vendor response constraints), with updated requirements feeding back into organizational playbooks and technical policy enforcement.

To make “real-time” explicit, the architecture is governed by latency budgets aligned to the evaluation metrics used in this study. Specifically, detection-to-isolation time reflects the combined latency of (i) sensing + anomaly detection at the grid edge and (ii) SOC triage/decision plus execution of isolation or automated containment. Likewise, recovery time reflects the latency of coordinated restoration actions across operators, EMS/SCADA control, and field crews, while patch/mitigation latency captures vendor-dependent remediation under governance constraints. In the framework-informed condition, the technical bundle (automated islanding, adaptive EMS setpoints, and edge anomaly detection) reduces the time-to-stabilize after manipulation events, while the organizational bundle (shared situational awareness dashboards and SOC–OT playbooks) compresses detection-to-decision time; governance mechanisms further reduce longer-horizon mitigation delays via federated threat intelligence and enforceable vendor timelines.

Table 7 summarizes the operational components and latency targets that enable real-time containment while separating longer-horizon governance actions from time-critical response.

**Table 7:**
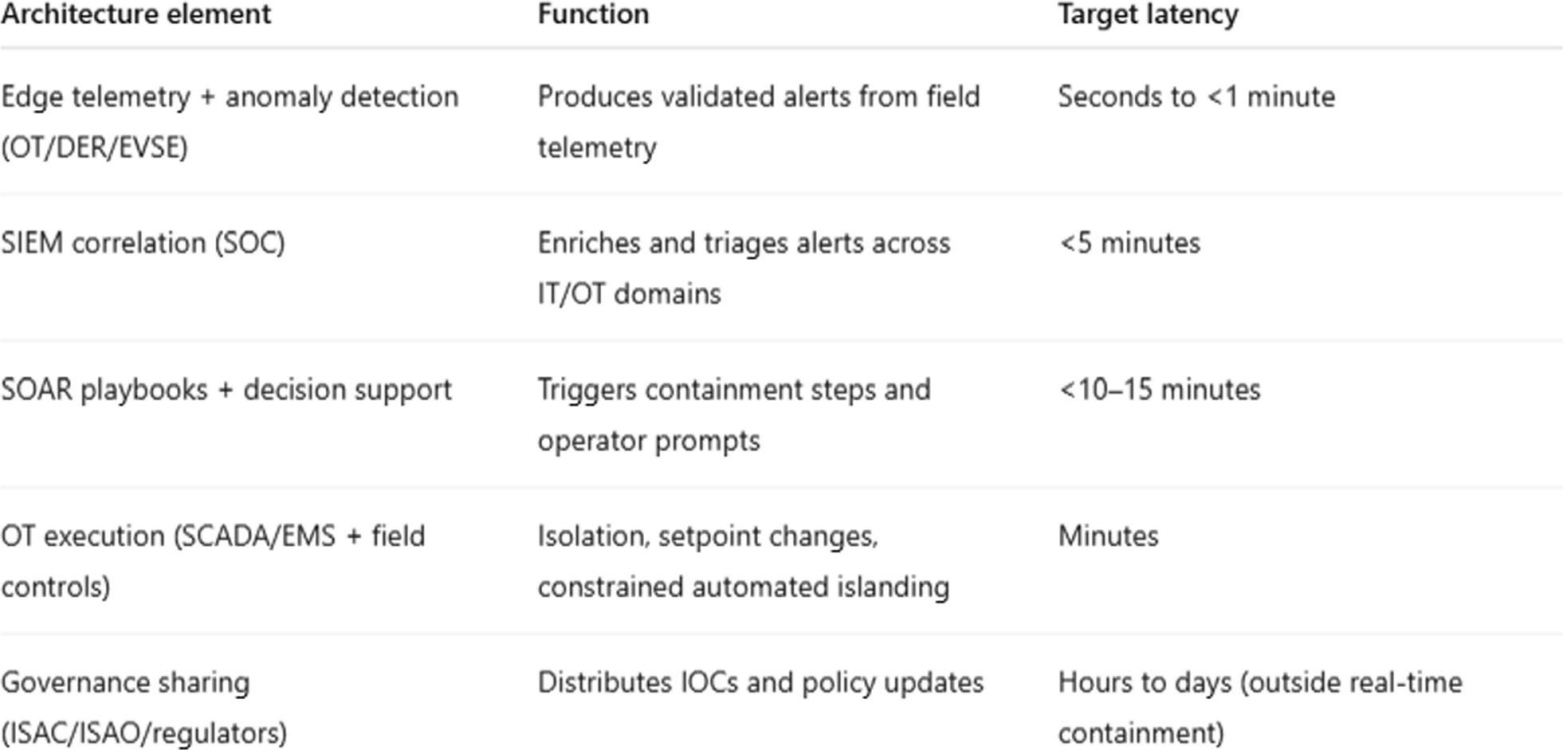
Real-Time Architecture Elements and Latency Targets (Framework-Informed)

Digital twin simulations further strengthen feedback loops by allowing stakeholders to test hypothetical scenarios, evaluate incident responses, and iterate policy and SOPs based on simulated outcomes (De Marco et al. 2025). Post-incident reviews, scenario-based drills, and structured debriefs build institutional memory and drive continuous improvement (CISA 2023).

These feedback mechanisms operationalize resilience engineering principles within STS theory, ensuring that resilience evolves with system complexity and external threats (Khan et al. 2025; Stouffer et al. 2023). This mirrors the STAMP principle that unsafe control can arise from delayed or distorted feedback at any level of the socio-technical hierarchy.

### Cross-layer integration and strategic relevance

The STAMP-adapted Socio-Technical Resilience Framework (Fig. 1) delivers a strategic, multi-layered defense model tailored for modern energy infrastructures. Unlike siloed security solutions, this approach embeds resilience into the interaction between technical systems, human processes, and governance mandates.

By coordinating detection, response, and recovery actions across layers, the framework enables energy operators to evaluate system-wide readiness and reduce the likelihood of cascading failures (i.e., prevent hazard paths shown in the sidebar of Fig. 1 from propagating). It supports a dynamic model of cybersecurity, one that adapts in real-time and learns from real-world disruptions.

Its practical relevance is validated in the next section, which applies the framework to two major incidents: the Colonial Pipeline ransomware attack and DER-related cyber disruptions in Europe. These cases demonstrate how gaps across the three layers contribute to systemic vulnerabilities and how strategic alignment can mitigate such risks.

## Case study analysis: colonial pipeline and DER-based grid disruptions

This section evaluates the applicability of the proposed socio-technical resilience framework by analyzing two critical cyber incidents:

1. The Colonial Pipeline ransomware attack (May 2021), and
2. Distributed Energy Resource (DER) grid disruptions in Europe (July–August 2022).

**Fig. 4:**
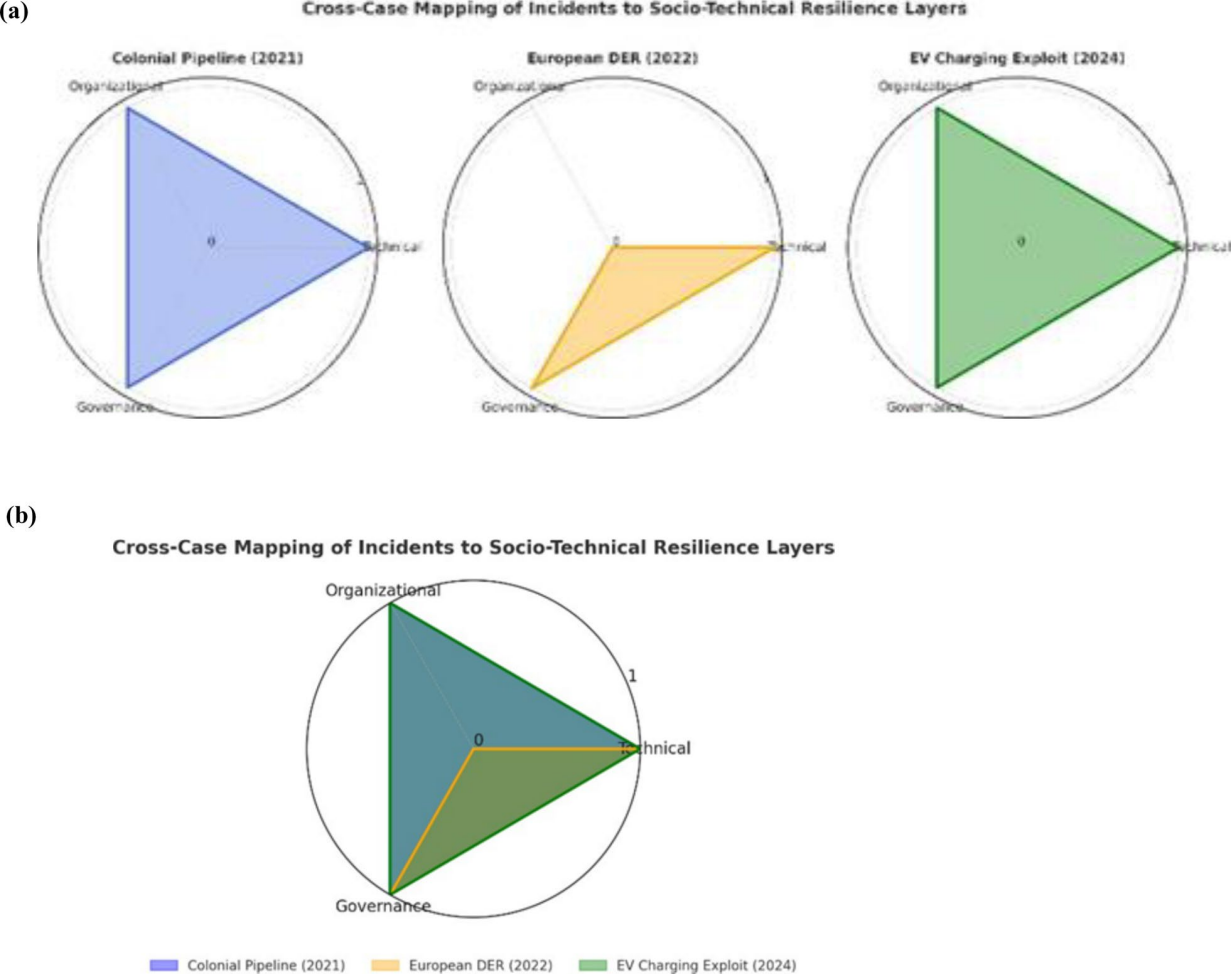
**A** (Individual Plots) (a) Individual radar plots by incident, color-coded for clarity, highlighting the distinct shapes of vulnerability across Technical, Organizational, and Governance layers. **B** (Combined Plot) Combined radar plot of cross-case mapping, showing overlapping socio-technical vulnerabilities across Colonial Pipeline (2021), European DER disruption (2022), and EV Charging Exploit (2024)

These cases highlight contrasting but complementary dimensions of vulnerability within hybrid energy systems. Applying a parallel structure across four analytical lenses (Failure Points, Framework Mapping, Response Gaps, and Implications) that enables a systematic cross-case comparison (see Fig. 4A, B).

### Colonial pipeline attack: organization and governance failures

#### Failure points

**Fig. 5:**
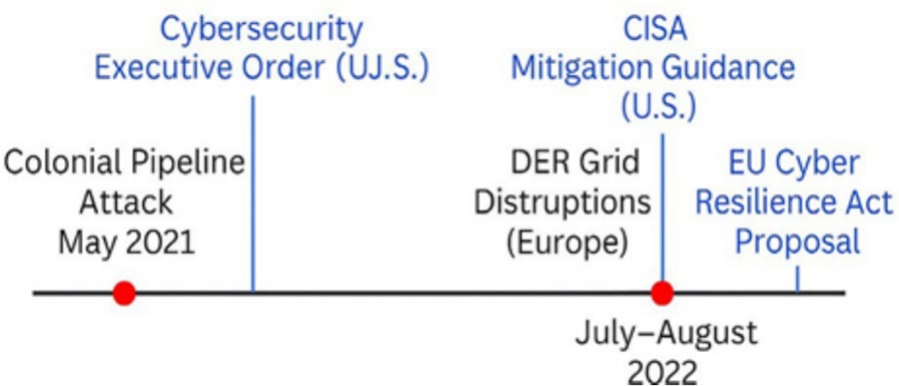
Timeline of two pivotal cyber incidents and related policy responses for hybrid grid security. The timeline situates the Colonial Pipeline ransomware attack (May 2021) and European DER disruptions (July–August 2022) alongside key responses, including the U.S. Cybersecurity Executive Order, CISA Mitigation Guidance, and the EU Cyber Resilience Act proposal

**Fig. 6:**
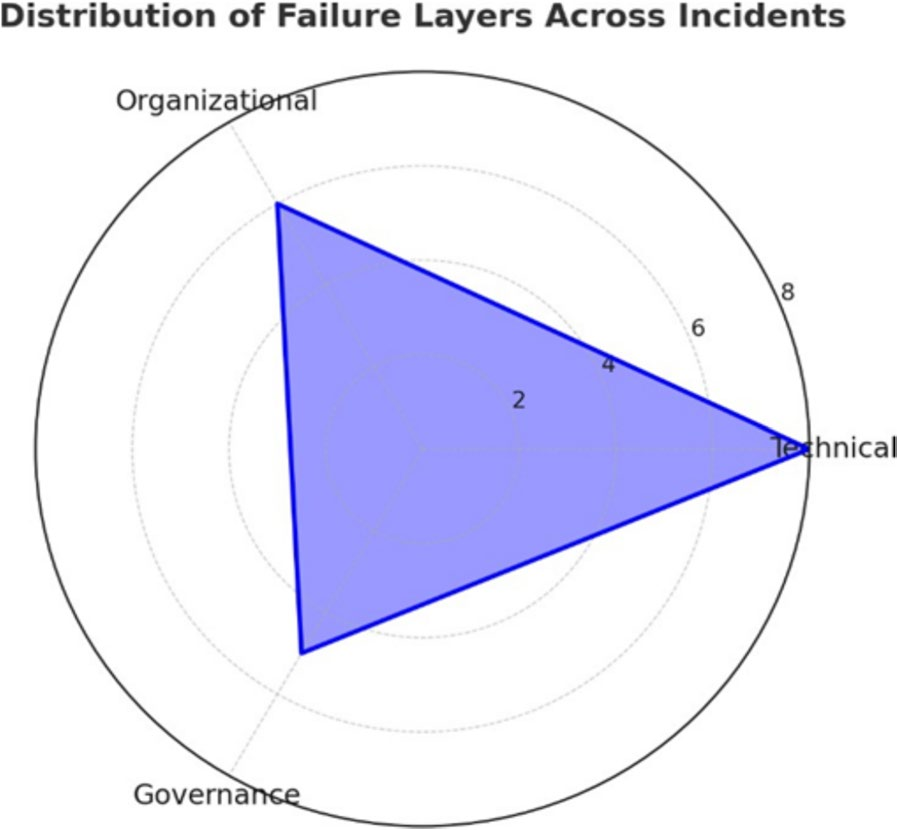
Radar plot of total incidents attributed to each socio-technical layer, showing Technical as most prevalent (8), followed by Organizational (6) and Governance (5)

In May 2021, a ransomware attack on Colonial Pipeline’s information technology (IT) systems prompted the company to proactively shut down its operational technology (OT) infrastructure, halting fuel distribution across the Eastern United States. At the time of the incident, Colonial Pipeline supplied approximately 45% of the East Coast’s fuel, and the shutdown resulted in widespread shortages and consumer panic across multiple states (U.S. Department of Energy [DOE], n.d.). Although the attack did not directly compromise physical pipeline systems, the decision to suspend operations reflected uncertainty-driven precautionary shutdowns arising from insufficient digital–physical coordination (Easterly and Fanning 2023). This event underscored vulnerabilities at the intersection of IT/OT governance, where limited situational awareness and fragmented decision support forced conservative actions with disproportionate national consequences (Lubin 2023). Similar patterns have been observed across the oil and gas sector, where targeted ICS and ransomware incidents increasingly exploit organizational and governance gaps rather than direct physical system access (Stergiopoulos et al. 2020) (Figs. 5 and 6).

### Incident-stage mapping to STAMP/STS framework domains

From a STAMP and socio-technical systems (STS) perspective, the Colonial Pipeline incident illustrates how cyber disruptions unfold through sequential stages shaped by control and feedback failures across technical, organizational, and governance layers. Rather than resulting from a single point of technical failure, the outage emerged through the accumulation of delayed feedback, incomplete process models, and unsafe control actions as the incident progressed from initial compromise to system-wide shut down. Mapping the incident into discrete stages clarifies how decision-making under uncertainty at organizational and governance levels amplified the operational impact despite the absence of confirmed OT compromise.

Table 8 maps each incident stage to the corresponding framework domain(s), identifies the relevant STAMP control or feedback failure, and highlights concrete technical, organizational, and governance interventions that could have reduced uncertainty and enabled more proportionate response actions.

**Table 8:**
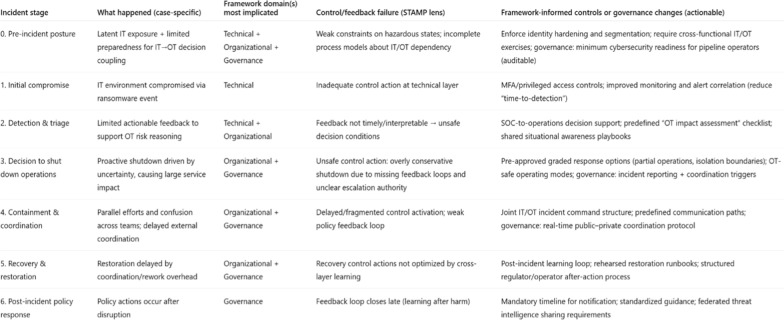
Colonial Pipeline incident stages mapped to framework domains and actionable interventions

This stage-based mapping demonstrates how classical STAMP constructs hierarchical control, unsafe control actions, process model gaps, and inadequate feedback which manifested across socio-technical layers during the Colonial Pipeline incident, revealing specific leverage points for resilience improvement.

### Controls, governance interventions, and potential impact

The framework highlights several control and governance mechanisms that could plausibly have mitigated the severity of the Colonial Pipeline outage. At the organizational level, predefined IT/OT escalation protocols, shared situational awareness dashboards, and decision-support tools tailored to OT impact assessment could have reduced uncertainty during early triage stages and limited reliance on full system shutdowns (Leveson 2003; Daida 2025). Joint incident command structures and rehearsed crisis simulations would further support safe control actions under time pressure.

At the governance level, enforceable cybersecurity readiness requirements for pipeline operators, standardized incident reporting timelines, and formalized public–private coordination mechanisms could have accelerated feedback between private operators and federal authorities, reducing delays in guidance and response activation (Shirtz et al. 2025). Treating governance explicitly as a STAMP controller clarifies how policy-level constraints and feedback timing shape operational decision-making during fast-moving cyber events.

While the precise counterfactual outcome cannot be measured directly, the framework’s quantitative evaluation provides an illustrative basis for estimating potential impact. In the discrete-event simulation, framework-informed interventions reduce detection-to-isolation time by approximately 44% and time-to-recovery by approximately 37% relative to baseline conditions (Table 4; Fig. 3). Applied conservatively to an incident such as the Colonial Pipeline shutdown, where outage duration was driven largely by coordination and decision latency rather than technical remediation, similar proportional reductions in response and recovery time would be expected to meaningfully reduce service disruption. Given Colonial Pipeline’s critical role in East Coast fuel supply, even partial reductions in outage duration could have mitigated downstream shortages and associated economic and societal impacts (U.S. Department of Energy, n.d.;).

### Implications: toward STAMP-aligned resilience strategies

The Colonial Pipeline case demonstrates that even when physical infrastructure remains uncompromised, systemic fragility can arise from gaps in control and feedback across socio-technical layers. The incident highlights the importance of pre-negotiated public–private response protocols, organizational structures that support safe control actions under uncertainty, and governance mechanisms that accelerate cross-layer feedback. By explicitly mapping incident stages to the adapted STAMP control architecture, this analysis reinforces the need for resilience strategies that integrate technical defenses with organizational coordination and governance oversight, reducing the likelihood that uncertainty-driven decisions translate into large-scale operational outages.

### der-based grid disruptions in Europe: technical layer gaps

#### Failure points

Several European countries experienced localized grid instabilities stemming from cyber intrusions into smart inverter firmware. Attackers exploited DER components, including rooftop solar controllers and home battery systems, by manipulating voltage settings and signal integrity. These edge devices are outside traditional utility control zones, and the attacks highlighted systemic weaknesses in grid edge cybersecurity and the visibility of decentralized assets (Telila et al. 2025; Atawi et al. 2022).

#### Framework mapping

The DER case mainly involves the technical layer of the framework. Firmware flaws, default credentials, and insecure patching led to vulnerabilities. Many devices lacked anomaly detection systems, making operators unaware of malicious activity until grid issues arose (Skrodelis et al. 2024). Additionally, inconsistent protocols and updating cycles among vendors hindered a unified technical response due to the absence of standardized communication protocols (Mohammed et al. 2022).

#### Response gaps

Many organizations often failed to detect or isolate the attacks early due to incomplete asset inventories and limited device telemetry. In many cases, utilities did not have authority or access over DER devices installed by third-party vendors, making coordinated mitigation efforts slow and fragmented. Additionally, existing cyber incident reporting structures did not account for non-utility actors, creating blind spots in intelligence dissemination (Gorman et al. 2024).

#### Implications

The DER disruptions demonstrate that technical weaknesses at the grid edge, such as insecure firmware, limited telemetry, and inconsistent update protocols, can create vulnerabilities that, if not addressed, can lead to significant and widespread consequences. Because these decentralized assets operate beyond traditional utility oversight, malicious manipulation can cause voltage instabilities, frequency shifts, or unbalanced loads that ripple across local and regional grids. The event illustrates the urgency of establishing secure-by-default firmware standards, vendor accountability, and shared security baselines across the DER ecosystem (U.S. Department of Energy [DOE] 2022). Furthermore, it calls for expanded regulatory oversight that includes third-party asset operators as integral stakeholders in grid cybersecurity planning.

### EV charging network firmware exploit

In 2024, metropolitan public transit authority experienced anomalous load spikes and unexpected session terminations across 60 Level 3 fast-charging stations serving its electric bus fleet. Forensic review revealed a firmware compromise in charger control modules, inserted through an unpatched vendor API used for remote diagnostics. At the technical layer, manipulated firmware caused chargers to intermittently misreport state-of-charge data and initiate unscheduled power draws, creating instability in the local distribution feeder. At the organizational layer, the transit authority’s operations team initially attributed the behavior to weather-related fluctuations, delaying escalation and allowing the attacker to persist for several weeks. At the governance layer, the absence of a mandated cross-sector reporting protocol meant that utility partners were notified only after voltage deviations triggered automated protection trips, disrupting both grid stability and bus fleet operations. This event underscores how EV charging systems inherit grid-edge DER vulnerabilities while adding transport-sector dependencies that amplify socio-technical risk.

Beyond grid instability, compromised EV charging infrastructure has wider socio-technical consequences. For example, metropolitan bus fleets can cause outages that could paralyze urban transit, have passengers stranded and disrupt daily mobility. Safety concerns definitely could become an issue if vehicles stall mid-route due to manipulating charging cycles. At the grid level, synchronized charger failures can overload feeders, potentially triggering blackouts. As a result, these failures can lead to both economic and reputational damage for transit agencies, while also weakening public trust in electric mobility. Unlike the Colonial Pipeline and DER cases, EV charging exploits uniquely bridge energy and transportation sectors, which magnifies their overall systemic impact.

*Cross-Case Synthesis* – Together, the Colonial Pipeline, European DER disruptions, and the EV charging network exploit validate complementary aspects of the proposed framework. The Colonial case emphasizes the governance and organizational dimensions, showing how policy misalignment and slow escalation exacerbate operational risk even when physical assets remain intact. The DER case underscores the technical dimension, revealing how firmware flaws and limited telemetry at the grid edge create vulnerabilities that propagate silently until operational instability occurs. An integrated socio-technical approach combining robust technical safeguards, trained organizational response, and proactive governance alignment would have addressed failure points in both incidents, demonstrating the value of a multi-layered resilience framework.

### Lessons learned and implications

The Colonial Pipeline ransomware attack and DER-based grid disruptions in Europe demonstrate that system resilience in hybrid energy infrastructures is not solely a function of technical controls, but rather a product of coordinated readiness across socio-technical dimensions:

- Technical layer: Both cases highlighted weaknesses in anomaly detection, firmware integrity, and secure interoperability between legacy and modern systems (Mohammed et al. 2022); Telila et al. 2025). In the DER case, limited device telemetry and patching gaps delayed incident response (Atawi et al. 2022), a pattern also evident in EV charging infrastructure where insecure firmware or default configurations can be exploited to disrupt local grid stability. In the Colonial Pipeline case, the lack of IT/OT integration contributed to precautionary shutdowns (Easterly and Fanning 2023).
- Organizational layer: Each incident exposed gaps in operator training, role clarity, and procedural readiness (McEvoy and Kowalski 2019). The absence of red-teaming, real-time simulations, and decision-support tools contributed to cognitive overload and delayed containment (Silva et al. 2022; Lubin 2023).
- Governance Layer: Delayed cross-sector coordination and fragmented regulatory protocols were evident in both cases (Shirtz et al. 2025; Telila et al. 2025). The lack of shared threat intelligence platforms and inconsistent vendor compliance left institutional blind spots that exacerbated the impact of the attacks (Department of Homeland Security [DHS] 2025); Cybersecurity and Infrastructure Security Agency [CISA] 2024). In addition, limited data sharing between vendors restricted collaborative visibility. Emerging techniques like federated learning could address this by enabling anomaly detection without exposing raw operational data (Steinmann et al. 2024).

These events reaffirm that resilience must be designed as an emergent property of integrated planning and adaptive feedback loops. Failure to synchronize the layers increases the risk of cascading impacts, even in the absence of direct infrastructure compromise. Moving forward, sector stakeholders must prioritize a defense-in-depth approach that fuses technical hardening with human and institutional preparedness (Khan et al. 2025; Stouffer et al. 2023).

Like DER fleets, they operate at the grid edge under fragmented vendor management and inconsistent oversight. Applying the socio-technical framework to EV infrastructure would address these weaknesses while capturing the unique interdependencies between transportation services and grid stability.

## Discussion: theoretical and practical implications

The quantitative scenario simulation complements the case-based validation by demonstrating measurable reductions in disruption time and service-impact proxies when the framework’s recommendations are applied. Importantly, improvements are driven not only by technical containment, but by reduced socio-technical latency (detection, triage, escalation, and coordinated recovery), reinforcing the paper’s central claim that resilience emerges from integrated technical, organizational, and governance controls.

This study demonstrates that the modified STAMP-based socio-technical resilience framework provides a more comprehensive diagnostic and design tool for hybrid oil-renewable energy systems than conventional independent models. By structuring technical, organizational, and governance dynamics within a single control-feedback hierarchy, the framework highlights how misalignments across layers can lead to unsafe control actions, delayed responses, and system-level failure even when physical infrastructure remains operational.

The Colonial Pipeline and European DER disruptions both illustrate how resilience is not solely determined by detection or containment, but by the capacity for cross-layer coordination under uncertainty. In both cases, failures in feedback loops between IT and OT, operators and interfaces, regulators and asset owners, amplified response delays. These incidents underscore the STAMP principle that accidents and disruptions emerge from flawed interactions, not isolated component failures.

Importantly, this research contributes to socio-technical theory by embedding dynamic control relationships (from STAMP) within STS system layers, thereby enabling a more actionable interpretation of resilience. Unlike traditional resilience frameworks, which treat human and policy dimensions as secondary to technical mitigation, the proposed model treats all layers as co-equal and interdependent. This represents a theoretical advancement in applying systems safety models to cybersecurity contexts in energy infrastructure.

Practically, this framework can serve as a tool for cyber-resilience auditing, systems design, and incident review. It is especially relevant in contexts where DER integration and regulatory fragmentation create complexity that exceeds the assumptions of linear or deterministic risk models.

Despite these constraints, the framework serves as a scalable, multi-layered blueprint for engineering resilience across digital energy systems. It bridges the gap between theory and implementation, enabling stakeholders to operate resilience as an emergent and iterative property, rather than treating it as a static set of protective controls.

To strengthen the operationalization and validation of the proposed socio-technical resilience framework, future research should prioritize the following areas:

- Field validation in live DER environments
  Deploy the framework in active distributed energy systems to assess its scalability, real-time adaptability, and integration with existing operational controls.
- Simulation via digital twins and live-fire scenarios
  Leverage digital twin environments and controlled attack simulations to test the framework’s responsiveness to emergent threats, feedback latency, and cross-layer coordination challenges.
- Governance interoperability and structural mapping
  Analyze how disparities in organizational hierarchies, jurisdictional authority, ad policy mandates affect system-wide control synchronization in federated and privatized energy sectors.
- Stakeholder Convergence and Data Architecture Design
  Explore methods to align utilities, regulators, and third-party vendors around shared security baselines, supported by interoperable platforms for real-time threat visibility and control signaling.
- Adaptive Deployment through Collaborative Feedback Loops
  Develop iterative implementation models that incorporate continuous feedback from multi-actor engagement, enabling dynamic refinement rather than relying solely on pre-defined blueprints.

## Recommendations for resilience implementation

This section outlines three focused design strategies to enhance socio-technical resilience across hybrid energy grids. Rather than presenting an exhaustive array of defensive mechanisms, the approach emphasizes principles that directly address systemic misalignments, fragmented decision-making, and evolving cyber threats.

### Implement zero-trust architecture across energy ecosystems

Zero-trust architecture (ZTA) is a foundational design principle that assumes no implicit trust within any part of the network. For hybrid grids involving centralized oil infrastructure and decentralized DER nodes, ZTA provides a scalable model to restrict lateral movement by malicious actors. Trust is continually evaluated based on user identity, device posture, and real-time risk telemetry.

To utilize ZTA in energy environments:

- Micro-segmentation should be used to isolate SCADA control zones from IoT-connected assets and vendor-managed components.
- Least-privilege access must apply across both human operators and autonomous agents.
- Identity-aware firewalls and policy enforcement engines can dynamically grant access based on real-time contextual analytics.

These controls reflect Department of Energy and NIST guidance for securing operational environments against lateral threats and supply-chain infiltration (U.S. Department of Energy [DOE] 2022; Stouffer et al. 2023; Davis 2021). Additional frameworks, such as the Cyber Resilient Design Framework for hybrid systems, further emphasize the need for layered defenses across OT and IT interfaces (Ackenhusen et al. 2024).

### Enable shared situational awareness among stakeholders

Cyber resilience in hybrid grids depends not only on technical safeguards but also on a shared operational understanding across stakeholder groups. Incidents like the Colonial Pipeline ransomware event exposed how misaligned visibility and disjointed response protocols prolong recovery.

Key steps include:

1. *Unified dashboards* that integrate IT and OT telemetry for real-time anomaly tracking visible to security analysts, operators, and executives alike.
2. *Cross-agency cyber drills* to strengthen synchronization between utility operators, regulatory bodies, and third-party vendors.
3. *Cognitive interfaces* that prioritize context-specific alerts reduce information overload and facilitate timely decision-making, especially when integrated with digital twin models to simulate threat escalation and response (Gebhard et al. 2025).

Shared situational awareness enhances coordination during critical events and reflects recommendations from resilience modeling literature and public–private cybersecurity guidance (Gorman et al. 2024; Lubin 2023; CISA 2023).

### Operationalize federated threat intelligence for grid interoperability

Hybrid energy systems typically operate in fragmented environments managed by multiple stakeholders. Centralizing threat data collection is often infeasible due to privacy, proprietary control, and latency constraints. Federated threat intelligence presents a viable alternative by enabling learning and response without raw data exchange.

Recommendations include:

- *Standardized metadata schemas* to facilitate secure exchange of indicators of compromise (IOCs) and anomalous behaviors.
- *Federated learning models* that allow decentralized systems to collaboratively train detection algorithms without exposing sensitive local data (Chen et al. 2025; Purohit et al. 2025).
- *Sector-aligned information-sharing platforms* developed in compliance with CISA and NERC interoperability standards.

By adopting federated intelligence, hybrid grids can overcome institutional silos and propagate defense insights across energy sectors, improving collective situational readiness (Stern and Becker 2019; Steinmann et al. 2024).

## Conclusion

As hybrid energy systems evolve by merging traditional oil infrastructures with renewable distributed energy resources (DERs), they introduce cybersecurity challenges that extend beyond conventional technical boundaries. This paper proposes a layered socio-technical framework that strengthens resilience across technical, organizational, and governance domains, grounded in STAMP and STS theory. Traditional approaches that focus narrowly on component vulnerabilities or linear attack chains prove insufficient in addressing the feedback loops, control gaps, and human-technical misalignments that characterize modern grid environments.

By leveraging real-world case insights, interdisciplinary modeling, and domain standards, this framework illuminates how failures propagate not just through code or hardware but through fragmented vendor coordination, policy ambiguity, and weak cross-layer controls. The three resilience recommendations (zero-trust architecture, shared situational awareness, and federated threat intelligence) are designed to operationalize this layered model by aligning real-time telemetry, access enforcement, and collective readiness across stakeholders.

Ultimately, building cyber-resilience in hybrid grids demands a shift from reactive defense to proactive systems thinking. This involves continuous feedback between control layers, integration of human factors into design, and regulatory coordination that anticipates disruptions before they cascade. The framework’s adaptability also positions it for application to EV charging networks, a rapidly expanding yet underscored component of the hybrid energy ecosystem. Securing these interfaces will be critical to safeguarding both grid resilience and sustainable transportation in the coming decade. As grid infrastructures evolve, so must the methods used to protect them not just with better tools, but with better frameworks that account for the full spectrum of socio-technical risk.
